# Implicit time-place conditioning alters *Per2* mRNA expression selectively in striatum without shifting its circadian clocks

**DOI:** 10.1038/s41598-018-33637-y

**Published:** 2018-10-19

**Authors:** Tenjin C. Shrestha, Karolína Šuchmanová, Pavel Houdek, Alena Sumová, Martin R. Ralph

**Affiliations:** 1 Department of Cell and Systems Biology, University of Toronto, Toronto, Ontario Canada; 2 Department of Neurohumoral Regulations, Institute of Physiology of the Czech Academy of Sciences, Prague, Czech Republic; 3 Department of Psychology, University of Toronto, Toronto, Ontario Canada

## Abstract

Animals create implicit memories of the time of day that significant events occur then anticipate the recurrence of those conditions at the same time on subsequent days. We tested the hypothesis that implicit time memory for daily encounters relies on the setting of the canonical circadian clockwork in brain areas involved in the formation or expression of context memories. We conditioned mice to avoid locations paired with a mild foot shock at one of two *Zeitgeber* times set 8 hours apart. Place avoidance was exhibited only when testing time matched the prior training time. The suprachiasmatic nucleus, dorsal striatum, nucleus accumbens, cingulate cortex, hippocampal complex, and amygdala were assessed for clock gene expression. Baseline phase dependent differences in clock gene expression were found in most tissues. Evidence for conditioned resetting of a molecular circadian oscillation was found only in the striatum (dorsal striatum and nucleus accumbens shell), and specifically for *Per2* expression. There was no evidence of glucocorticoid stress response in any tissue. The results are consistent with a model where temporal conditioning promotes a selective *Per2* response in dopamine-targeted brain regions responsible for sensorimotor integration, without resetting the entire circadian clockwork.

## Introduction

Animals in nature gain significant adaptive advantages in terms of risk avoidance or energy conservation by anticipating important conditions (e.g. food availability, potential predation), at times when an encounter with such conditions is most likely. Whereas the ubiquitous light-entrainable circadian clocks enable animals to prepare for the regular daily changes in environmental conditions, the anticipation and response to transient, day-to-day events, requires a memory of when significant events have been encountered before. Time memory has been demonstrated in a broad range of species including bees^1–3^, birds^4^, rodents^5–7^, and primates^8^.

The time of day can be remembered by organisms either explicitly or implicitly. When presented explicitly, the ‘time’ is registered as a unique feature of the environment that discriminates when an encounter occurs from another time when the condition is absent. This requires associative conditioning of a neural representation of the time of day. In this way organisms may use time as a discriminative cue (a conditioned stimulus) to recognize whether or not the event or condition (an unconditioned stimulus) is likely to occur. Alternatively, implicit time memory is inferred in situations when animals have no information with which to compare conditions at more than one time of day. In this case, memory for time of day is indicated when responses can be elicited only within a restricted few hours near the time of the original encounter. Implicit time memory produces non-cognitive responses that require the setting of condition entrainable circadian oscillators (CEOs) at the time of an event. In this way the possible recurrence of an event is anticipated automatically at 24 hour intervals, without the need for experience with a conditioned context at other times^9^.

Our current view of implicit time memory is based on evidence that animals register the time of day by using reference signals generated by a circadian oscillator that is synchronized with the timing of biologically significant events^5–7^. This model expands on earlier demonstrations that even single encounters with significant conditions could create periodic response deficits^10–13^. The master circadian clock in the suprachiasmatic nucleus (SCN) is not required for time memory. Rodents with SCN lesions exhibit daily performance peaks that are set to the time of conditioning on several learning paradigms including conditioned place avoidance (CPA), conditioned place preference (CPP), and passive avoidance (PA)^14–16^. Furthermore, Syrian hamsters homozygous for the mutation, *tau* (*Ck1ε*^*tau/tau*^), continue to express 24-hr modulation of CPA response despite their molecular clocks producing 20 hours rhythmicity in their behavior^17^. These data indicate that the CEOs register time using mechanisms that are distinct from the circadian clocks controlling inherent daily rhythms of behavior. Nevertheless, although the clock in the SCN is not necessary for time memory, its signals may act as a *Zeitgeber* (external timing cue), producing circadian entrainment of a CEO underlying previously conditioned anticipatory behavior^7^. Time memory therefore, is a function of the circadian hierarchy that enables organisms to anticipate significant conditions and events based on both the long term (regular) and short term (transient) likelihood of their occurrence.

The anatomical locations and circadian mechanism underlying time memory are currently unknown. A diverse body of literature suggests that the acquisition of a time memory depends on dopamine activity. Dopamine is involved in the establishment of context memories including reward learning^18,19^ and responses to aversive stimuli^20–25^. More specifically, anticipatory behavior in animals held on restricted feeding schedules, which require a memory of food availability, is dopamine dependent^26,27^. A CPP can be produced by amphetamine administration^28,29^, and time-place memory associations in hamsters have been shown using amphetamine and haloperidol in CPP and CPA tasks respectively^30^. Furthermore, established time-place avoidance in hamsters may be reset to a different time using single injections of amphetamine^30^. Together, these results suggest that a clock underlying time memory is associated with one or more of the ascending dopamine arousal systems including dopamine sensitive brain targets.

The ascending dopamine systems in the brain comprise the nigrostriatal, mesolimbic, and mesocortical pathways. Therefore, it is possible that numerous temporally linked pathways transmit the timing of an event. Although the central circadian clock in the SCN is not required to establish time memory^17^, peripheral molecular circadian clocks are active throughout the brain^31,32^, and in many regions shift their timing in response to conditioning stimuli (e.g. fear/anxiety^33,34^, scheduled food availability^35–37^). To identify possible neural substrates responsible for time memory, we examined clock gene expression in major neural targets of dopamine projections to determine whether the phases of region specific molecular clocks were affected by place avoidance conditioning.

**Figure 1:**
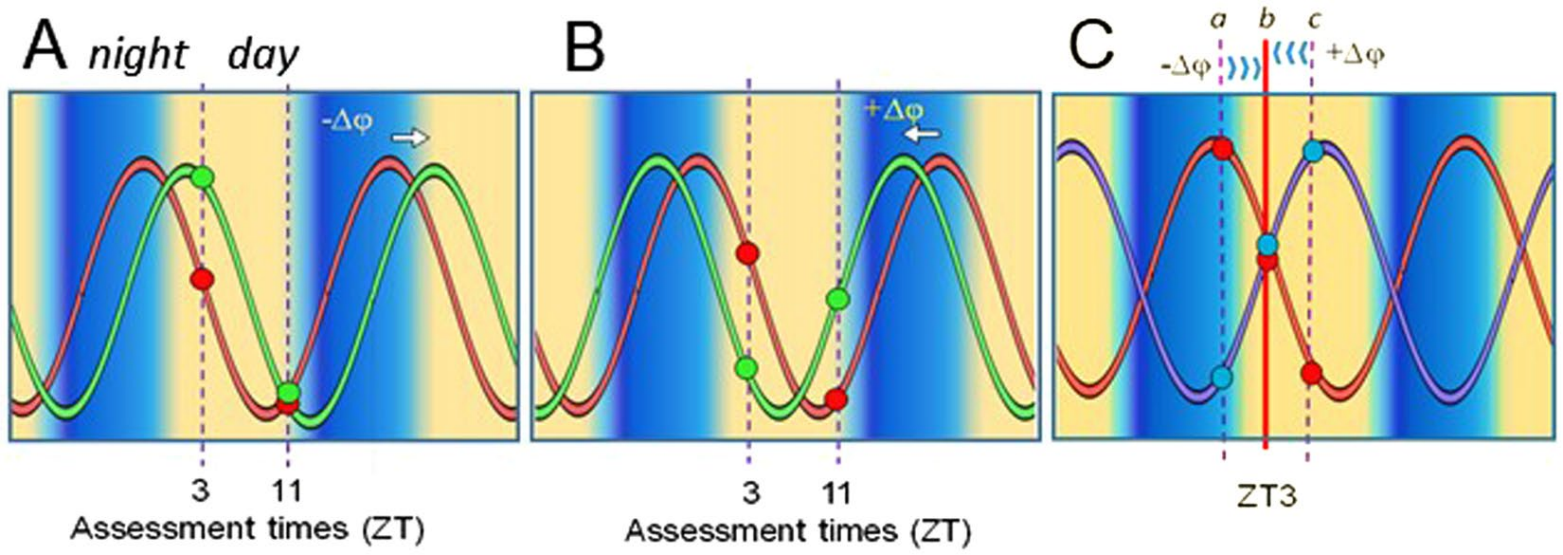
Model for detection and validation of phase resetting responses. (**A,B**) Phase resetting of a single, stable oscillating variable (shown in red) to an advanced position (**A**) or a delayed position (**B**) (shown in green) relative to an external time cue or Zeitgeber. Changes in phase angle (in entrained systems) or phase shifts in freerunning systems is detected by comparing values at two Zeitgeber (ZT) or circadian time points. Measurements at single time points may correlate with phase but are imprecise due to variability, and are confounded in that more than one point may be associated with the same value. The state of an oscillation is represented more accurately by a combination of corroborating values at two *Zeitgeber phases* (e.g. ZT3 and ZT11 in the model). Additional points may be necessary to account to include to control for acute amplitude changes that may give false positive results or for when measurements occur near the nadir or acrophase that may give false negatives. (**C**) Assessment of phase resetting using multiple state variables. A stable circadian cycle, whether entrained or freerunning, predicts that reliable state variables in the molecular cycle will attain relatively stable phase relationships, and will return to these relationships after a resetting of the overall cycle. Assessment of a stable component (shown in red) is accompanied by a second clock component (shown in purple). Resetting of the oscillator system is indicated by predictable changes in the ratios of the two components when measured at different phase reference points. Therefore measurements taken at ZT3 (b, as shown), should reflect the ratios at the points indicated by the vertical dashed lines (a or c), if a phase shift has occurred.

In most experimental paradigms stable phase resetting of an oscillator requires sampling multiple cycles following a stimulation. However, time memories are acquired rapidly following an encounter^17^, and must be fully established within 24 hours to provide the greatest benefit to the organism. Furthermore, for animals in nature the advantage of anticipating when important conditions might recur, suggests that multiple time memories can be held simultaneously. For these reasons, we looked for the initial perturbation of clock gene expression in the first 24 hours following a conditioning protocol, testing the hypothesis that the creation of a time memory involves the rapid resetting of a molecular clockwork that may be required for the processing of future events. The experimental protocol is based on a corroboration model of resetting wherein alterations of a single state variable (clock gene) at one phase will predict changes in that variable at another phase, as well as changes in other state variables. The model is described graphically with further explanation in Fig. 1.

## Results

### *In situ* hybridization (ISH) and qRT-PCR sensitivity to phase in the SCN

**Figure 2:**
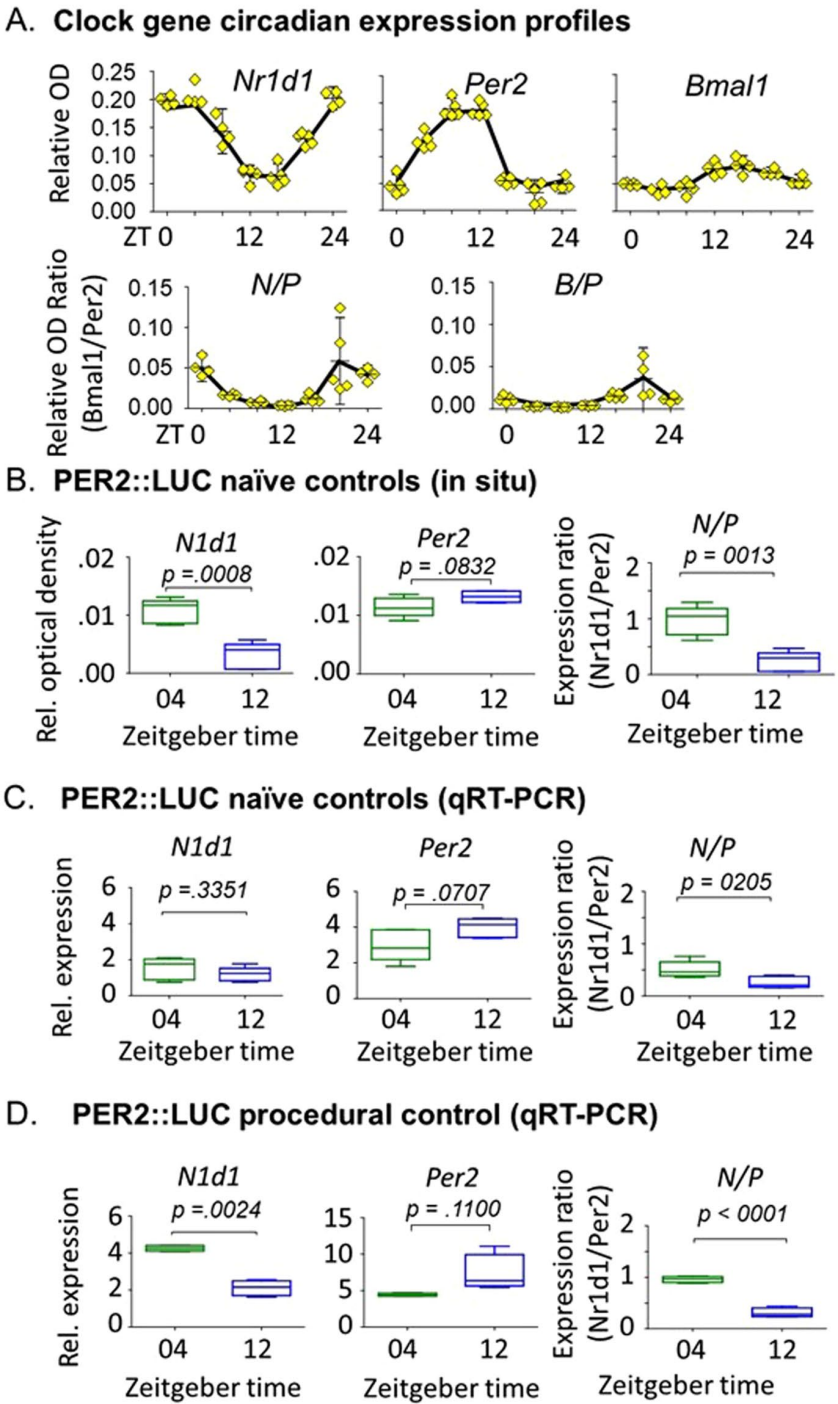
Temporal expression of circadian clock genes in the SCN. (**A**) 24-hour temporal expression of *Nr1d1, Per2*, and *BMAL1* mRNA measured *in situ* in the SCN relative to a region of similar size, dorsal and lateral to the SCN. Assessments were relative to GAPDH. N/P and B/P represent the within subject expression ratios of *Nr1d1*/*Per2* and *BMAL1*/*Per2*, respectively. Error bars = SEM. Data are from C57Bl6 mice; n = 5 per group. (**B–D)** Relative mRNA expression of *Nr1d1*, *Per2*, and N/P ratio at ZT04 and ZT11 in two control groups, using two mRNA quantification methods. P values are based on individual t-tests. N = 4/group.

We ensured first that measurements of clock gene transcripts were sufficiently sensitive and reproducible to allow the detection differences in circadian phase as well as small changes in expression indicative of a stimulated response or phase resetting. In the first experiment, using wild type C57Bl6 mice, we determined daily mRNA profiles of three clock genes, *Per2, Nr1d1, and Bmal1* using ISH. Twenty-four hour expression profiles from unconditioned control mice are shown in Fig. 2A. From these we derived daily profiles for the ratios *Nr1d1*/*Per2* (N/P), and *Bmal1*/*Per2* (B/P) in selected regions, to be used as confirmatory indicators of phase or phase shifts. From the expression profiles, we chose ZT3 and ZT11 for conditioning, and ZT4 or ZT12 for behavioral testing and gene expression assessments at matching or nonmatching times. These times bracketed part of the circadian dynamic ranges of expression for the three clock genes, and avoided confounding effects of acute light/dark (LD) changes.

In the second experiment, we confirmed that predicted phase dependent differences in single gene expression in the SCN (as shown in Fig. 2A) as well as gene expression ratios, were present in both naïve and procedural controls. A consistent pattern of *Nr1d1* and *Per2* expression was found for both unconditioned control groups, and both techniques used to assess the transcript levels (ISH; Fig. 2B and qRT-PCR; Fig. 2C,D). *Nr1d1* levels were higher at ZT04 than at ZT12, and *Per2* levels were higher at ZT12 than at ZT04. The N/P ratio was always significantly higher at ZT04 than ZT12, confirming the phase dependent expression obtained from the ISH experiment using wild type controls (Fig. 2A). In qRT-PCR measurements, both *Nr1d1* and *Per2* levels at both time points were higher in procedural controls than naïve controls (Fig. 2C,D), suggesting an effect of experience. Therefore, the procedural controls were used in experiments for assessing effects of conditioning.

### CPA and implicit time memory

**Figure 3:**
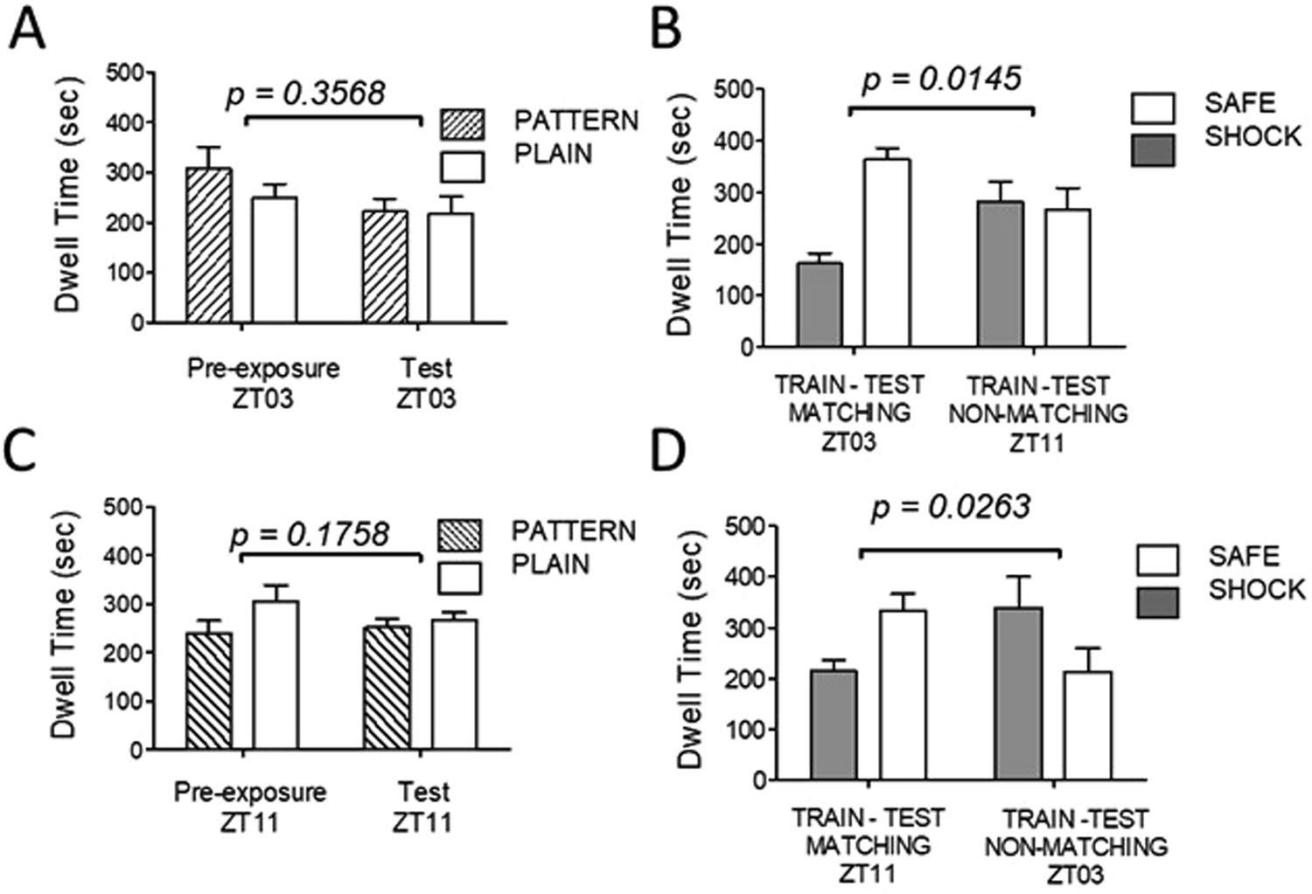
Time memory in avoidance conditioning. (**A**,**B**) Place avoidance conditioning at ZT03. (**C**,**D**) Place avoidance conditioning at ZT11. Animals were pre-exposed to the conditioning chambers to reduce interference due to situation novelty. Control animals at ZT03 and ZT11 (**A,C**) showed no preference for either conditioning chamber during pre-exposure and testing 8 days later. Data represent average dwell times in the striped and plain context chambers indicated. Conditioned animals at ZT03 and ZT11 (**B,D**) showed significant avoidance of a shock-paired chamber after 8 days of conditioning. Avoidance was expressed when testing occurred at the matching time, but not at the non-matching time. (Non-conditioned, N = 4/group; conditioned, N = 5/group). P values are associated with the difference in dwell time in the shock-paired chamber versus the safe chamber (t-test). Error bars = SEM.

Unconditioned animals were passed through the conditioning paradigm at either ZT03 or ZT11 without foot shock, and were tested for preference after eight days at either matching or non-matching times. Control animals showed no significant preferences for context during habituation (Pre-exposure) nor testing (Test). All animals visited each context chamber at least twice during both habituation and testing (Fig. 3A,C). A second cohort of animals was passed through the conditioning paradigm, receiving the foot shock stimulus in one of the two context chambers at either ZT03 or ZT11. Mice were tested at either matching or non-matching times on the day after the final conditioning trial (Fig. 3B,D). Following conditioning, animals trained at ZT03 avoided the shock chamber when tested at ZT03 (p = 0.0003), but not when tested at ZT11 (p = 0.7948; Fig. 3B). Animals trained at ZT11 significantly avoided the shock chamber when tested at ZT11 (p = 0.0458), but not at ZT03 (p = 0.1446; Fig. 3D). Difference scores were compared to determine whether group preferences were changed after conditioning. Preferences between contexts did not change at either ZT03 or ZT11 in unconditioned controls, however, they were significantly higher at the matching time following conditioning at either ZT03 or ZT11.

### Effects of conditioning on temporal expression of Nr1d1 and Per2

**Figure 4:**
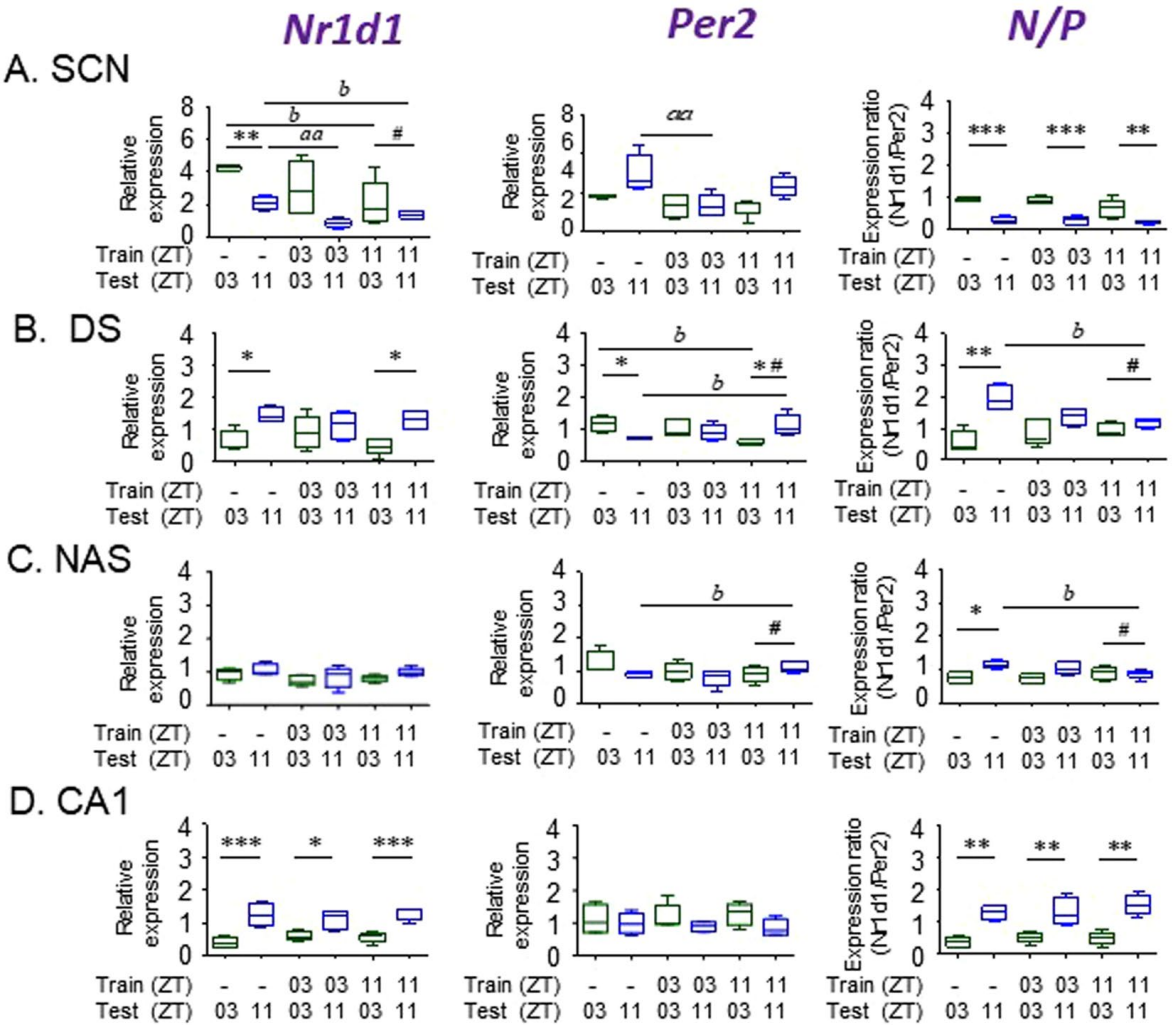
Effects of behavioral conditioning on temporal gene expression. *Nr1d1, Per2* and N/P ratio is shown in four selected tissues. Each graph represents two conditioning experiments comparing gene expression following place conditioning at either ZT03 or ZT11 with procedural controls. Animals were tested for place preference at either ZT03 or ZT11. Tissues were obtained one hour after testing. Phase dependent differences within each experimental condition (control, ZT03 conditioned, ZT11 conditioned) were evaluated using planned comparisons and t-tests [^*^p < 0.05; ^**^p < 0.01, ^***^p < 0.001]. Effects of conditioning were evaluated using one-way ANOVAs for control vs. ZT03 conditioning [^*aa*^*p* < 0.01] and vs. ZT11 conditioning [^*b*^*p* < 0.05; ^*bb*^*p* < 0.01]). Alteration in the relative expression at ZT03 vs. ZT11 were determined from difference scores between the two times [^#^*p* < 0.001]. *P* values for comparisons of all difference scores are shown in Table 1. Error bars = SEM.

We determined the effect of place avoidance conditioning on the expression of *Nr1d1* and *Per2*, and on the N/P ratio in the SCN, DS, Ci, hippocampus (DG, CA3 and CA1), the nucleus accumbens shell (NAS) and core (NAC), the basolateral amygdala (BLA), and central nucleus of the amygdala (CeA). We compared gene expression in control animals (tested at ZT03 or ZT11 without conditioning) with animals conditioned at either ZT03 or ZT11 prior to testing at either matching or not-matching ZT using qRT-PCR. Results obtained for selected brain areas (SCN, DS, NAS and CA1) are shown in Fig. 4. These areas were selected for specific reasons: Although the SCN is not required for time memory, it functions as a source of circadian entrainment signals for the CEO underlying time memory. Also, nonphotic arousal associated with conditioning and testing, could be relayed via changes in SCN activity. In the DS and NAS, significant changes in gene expression following conditioning suggest a shift in the molecular clock. Finally, although post-conditioning effects were not significant the CA1, gene expression represents the pattern expected from tissue clocks that do not respond conditioning. Results from Ci, DG, CA3, NAC, BLA and CeA were similar to CA1, and are shown in Supplementary Fig. S1. Results from the qRT-PCR study were confirmed using ISH from control animals, and are presented in Supplementary Figs S2–S6. The absence of response in most regions indicates molecular circadian clocks are not likely to be involved in time memory in those tissues. Details of findings by area are as follows:

#### SCN

In control animals, *Nr1d1* mRNA was significantly higher at ZT03 than ZT11 using qRT-PCR (*p* = 0.0066) and ISH (*p* = 0.0008). Following conditioning at ZT03 the phase dependent difference in expression was not significantly affected, but the difference was eliminated after conditioning at ZT11. *Per2* expression was higher at ZT11 in controls, but the difference was not significant using either qRT-PCR (*p* = 0.1100) or ISH (*p* = 0.0832). Following conditioning at ZT03, *Per2* was reduced at ZT03 (p = 0.0471), thereby eliminating the small phase dependent difference. The phase dependent difference was unaffected by conditioning at ZT11. Despite these significant main effects of conditioning on both *Nr1d1* and *Per2*, there were no significant conditioning effects on N/P ratios at either ZT03 or ZT11, and no changes in the phase dependent difference (ZT03 vs. ZT11) between controls and either conditioned group (Fig. 4A).

#### Dorsal striatum

In control animals the expression of *Nr1d1* and *Per2* were both significantly different between ZT03 and ZT11 (p = 0.0113 and p = 0.0371, respectively). This was confirmed by the ISH data (Supplementary Fig. S2). *Nr1d1* expression was higher at ZT11 than ZT03, while *Per2* levels were higher at ZT03. Phase dependent differences in mRNA levels for both genes were reversed relative to the SCN values. The phase dependent difference in *Nr1d1* expression was eliminated following conditioning at ZT03, while conditioning at ZT11 had no effect. Phase dependent *Per2* expression became non-significant following conditioning at ZT03. Conditioning at ZT11 resulted in a significant time dependent difference in expression that was reversed relative to controls (p = 0.0159). N/P ratios were phase dependent in controls (p = 0.0029), again in reverse temporal order compared with the SCN. Following conditioning at ZT03, the main effect of test time on N/P dropped to p = 0.1233. This was due to a combined increase at ZT03 and decrease at ZT11 neither of which were significant alone (p = 0.4653 and p = 0.2279, respectively). A greater effect on N/P was produced following conditioning at ZT11. Overall, the effects of conditioning on N/P at ZT03 were due to changes in *Nr1d1*, whereas effects at ZT11 were due to significant changes in *Per2* expression. Importantly, a combination of the main effects on *Per2* expression resulted in a highly significant change in the difference between these two test times (p = 0.0016 for *Per2*; p = 0.00015 for N/P; Table 1). Therefore, in the DS, the phase dependent effects of conditioning on rhythmic gene expression were exhibited mainly by changes in *Per2* transcription, which were amplified when integrated with lesser effects on *Nr1d1* produced at the opposite ZT. A full analysis of changes in difference scores (ZT03 minus ZT11) is presented in Table 1 and discussed below (Fig. 4B).

**Table 1:** Effects Of Conditioning On Phase Dependent Expression Of Clock Genes.

Brain area	Conditioning time (ZT):	*Nr1d1 P*=	*Per2 P*=	*N/P P*=
SCN	03	0.9835	0.0472	0.5305
	11	0.0612	0.7331	0.1677
Cingulate	03	0.0192	0.5553	0.9701
	11	0.0416	0.0469	0.8183
Dorsal Striatum	03	**0.0054**	0.072	0.0215
	11	0.9828	**0.0016**	**1.50E-04**
Dentate	03	0.1046	0.0132	0.9883
	11	0.7688	0.9635	0.6803
CA3	03	0.8939	0.1362	0.3958
	11	0.5093	0.0839	0.2396
CA1	03	0.0318	0.2273	0.4758
	11	0.1868	0.0772	0.0528
N. acc. Shell	03	0.9708	0.2985	0.1856
	11	0.1996	**0.0016**	**2.38E-05**
N. acc. Core	03	0.7883	0.1312	0.5115
	11	0.7296	0.1337	*0.0068*
Lat. Amygdala	03	0.0598	0.0808	0.8386
	11	0.193	*0.0082*	0.4838
CN amygdala	03	0.7249	0.9802	0.9323
	11	0.1594	0.1913	0.014

Probability values (*P*) indicate the degree to which the temporal difference in gene expression between controls at ZT03 and ZT11 has changed following conditioning. Numbers in **bold** type indicate changes that meet the *P* < 0.01 likelihood that this is not a random occurrence based on statistical difference and is supported by corroborating changes at other time or genes. Numbers in *italics* indicate significant changes that are not corroborated by changes at other times or in other genes.

#### Nucleus accumbens shell (NAS)

Qualitatively, the NAS resembled the DS in the responses of both *Nr1d1* and *Per2*. Neither *Nr1d1* nor *Per2* phase dependent differences were significant in controls, but the combination, N/P, reached significance. Following conditioning at ZT03, *Nr1d1* levels were not altered at either test time. However, following conditioning at ZT11 *Per2* expression levels were significantly higher at the matching time than nonmatching time. Not surprisingly the N/P ratio did not change significantly following conditioning at ZT03, but was reduced following conditioning at ZT11. As with the DS results, conditioning at ZT11 was followed with a significant change in the difference of response at ZT03 and ZT11. Considering *Per2* alone, the difference between testing at ZT03 and ZT11 were altered significantly (*p* = 0.0016), and the N/P ratio was changed significantly (*p* = 0.0000238; Table 1) (Fig. 4C).

#### Other brain areas

Other brain tissues examined, hippocampus (DG, CA3, CA1), Ci, NAc, BLA, CeA, did not respond to conditioning at either ZT). Assessments of gene expression from the CA1 are presented for comparison with tissues where gene expression was altered by conditioning as an example of the patterns expected if conditioning had no effect (Fig. 4D). In the control CA1, *Nr1d1* expression was significantly higher at ZT11 than at ZT03 (*p* = 0.0142), whereas *Per2* was not significantly different at the two times (*p* = 0.7005). The N/P ratio between the two times in controls (*p* = 0.0015), and the phase dependent difference remained significant following conditioning at either ZT03 (*p* = 0.0078) or ZT11 (*p* = 0.0004) (Fig. 4D and Supplementary Fig. S1).

In the remaining brain regions examined, expression patterns of the two genes were similar to the CA1 (Supplementary Fig. S1). In control animals, *Nr1d1* was consistently higher at ZT11 than at ZT03, but this difference was only significant in DG (p = 0.0093) and CA3 (0.0414). *Per2* did not exhibit phase dependent differences in any of these brain regions. However, N/P ratios exhibited significant phase dependence in all control groups except amygdala (BLA, CeA). The amygdala showed low levels of clock gene expression, with no circadian rhythms in controls and no rhythmic expression following conditioning (Supplementary Fig. S1). Gene expression in the Ci was qualitatively similar to that of the hippocampus. Expression of *Nr1d1* was higher at ZT11 than at ZT03 in all groups, although none of the comparisons reached significance. For *Per2*, only conditioning at ZT11 had a significant effect on testing at ZT03 (p = 0.0107). The N/P ratio for non-conditioned controls was weakly significant between ZT03 and ZT11 (p = 0.0486) and was unaffected by conditioning at any ZT. In the amygdala, there were no significant differences between ZT03 and ZT11 for either gene or N/P ratio.

### Effect of conditioning on *Bmal1* expression

**Figure 5:**
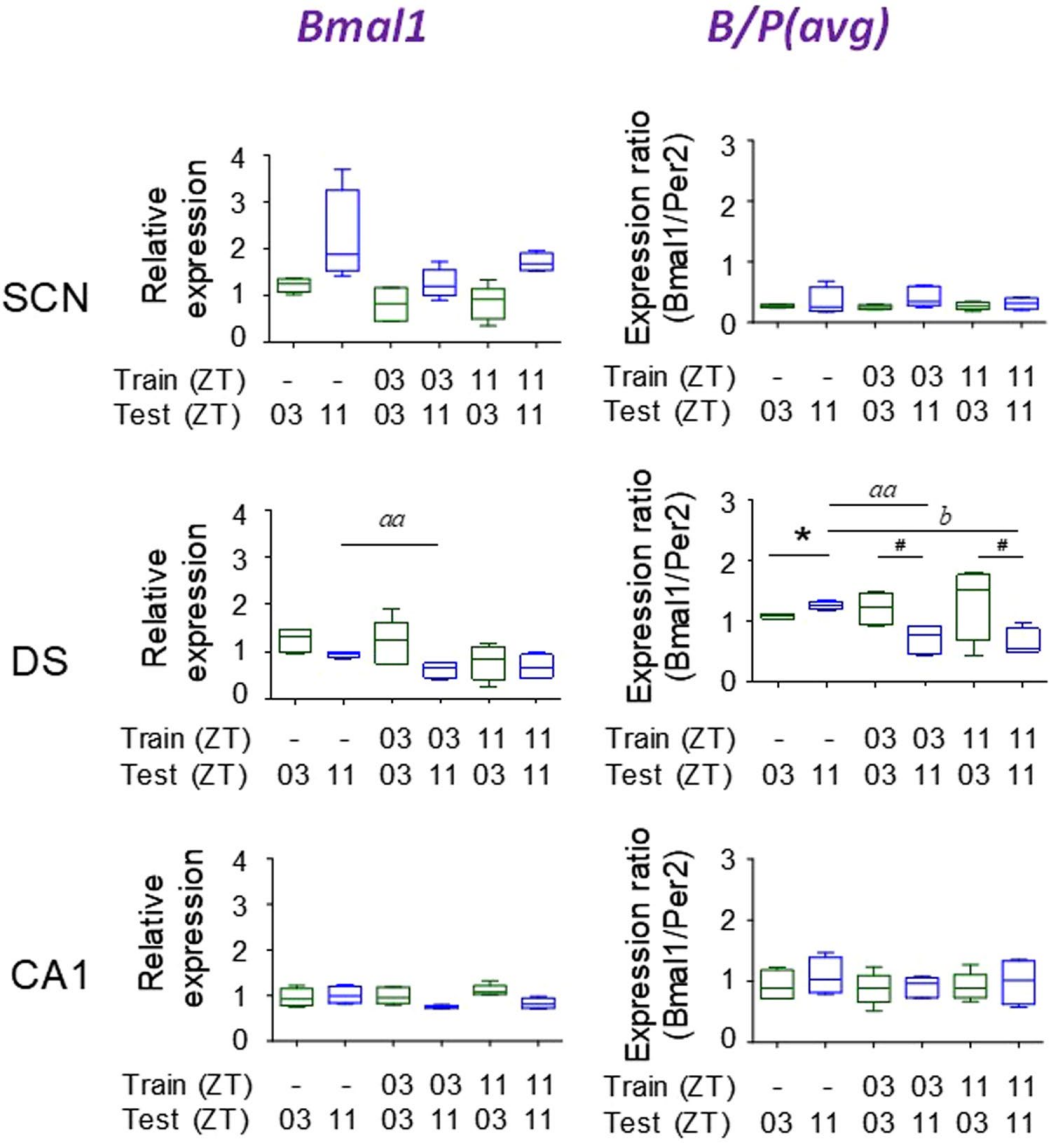
Effect of behavioral conditioning on temporal gene expression of *Bmal1*. Expression of *BMAL1* in SCN, DS, and CA1. Training and testing times are indicated on each graph (see Fig. 4). [N = 5/group; ^*^p < 0.05; ^*aa*^p < 0.01, ^*b*^p < 0.05]. ^#^Significant change in difference scores versus controls. [*p* < 0.001; N = 4/group]. Error bars = SEM.

To further probe suggested phase shifts in the SCN, and DS, we extended the data by assaying *Bmal1* expression by qRT-PCR in these brain areas. CA1 was used as a possible negative control, as tissue changes in *Bmal1* expression are not predicted from the responses of *Nr1d1* and *Per2*. A possible effect of conditioning on *Nr1d1* in the SCN, had been suggested previously (Fig. 4A). No significant phase dependent differences were found for either *Bmal1* expression or B/P in either SCN or CA1 (Fig. 5A,C), and neither *Bmal1* expression nor B/P were affected by conditioning. Together with the absence of any obvious change in *Nr1d1* or N/P ratio due to conditioning, the *Bmal1* data support the conclusion that clock resetting has not occurred in the SCN nor CA1. The results were different for *Bmal1* in the DS. *Bmal1* expression was reduced following conditioning at both ZT03 and ZT11, but was significant only for the ZT03 conditioned group (Fig. 5B). The effect of conditioning was amplified in the B/P results. The changes in B/P are likely to be explained as a combination of reduced *Bmal1* expression with the increased *Per2* expression relative to controls (cf. Fig. 4B).

### Comparison of qRT-PCR data using two different reference genes

Appropriate reference genes for qPCR have been the subject of considerable discussion and review^38–40^. Some genes are problematic due to variability, differences in expression across tissues, and/or sensitivity to changes in cell function. GAPDH is used widely as a useful reference in studies of brain tissue, and is generally accepted as a stable reference for sleep and circadian rhythm studies^41–43^. Despite being linked to cell metabolism, GAPDH expression has been shown to be stable over time, and an appropriate reference for studies of rhythmicity in the brain. In our own studies we have found GAPDH expression in brain tissues to be stable over time. We added B2M as a comparative reference in order to test for rhythmic and/or conditioned changes due to conditioning. B2M is part of a different cellular process from GAPDH, and the two should be subject to separate regulation in the temporal cellular environment. We found no differences in the temporal expression of clock genes relative to the two HKGs. Data comparing GAPDH and B2M as reference genes are shown in Supplementary Fig. S7.

### Glucocorticoid and cFOS responses to conditioning

**Figure 6:**
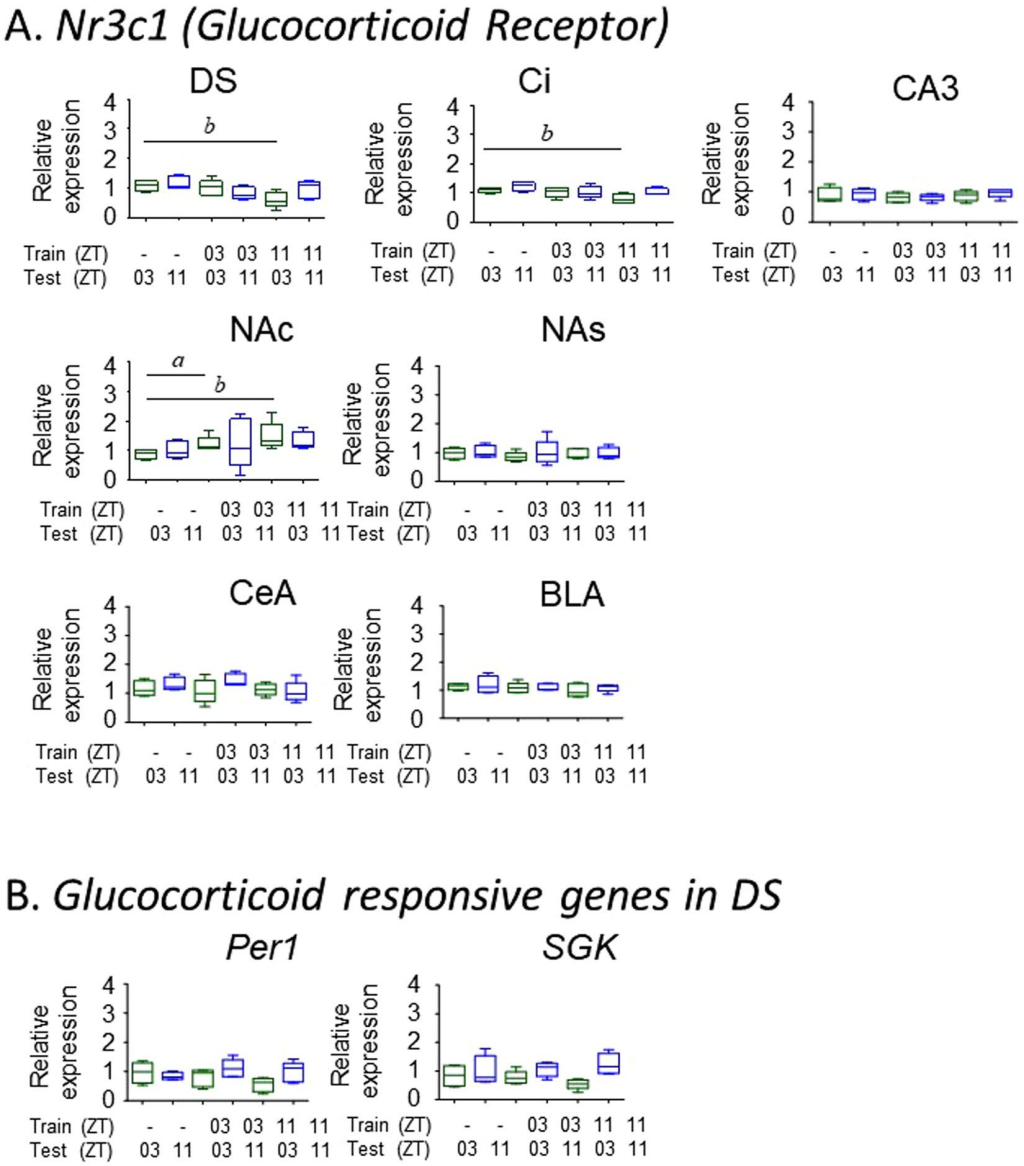
Effect of behavioral conditioning on glucocorticoid responses. (**A**) Expression of the glucocorticoid receptor in various brain tissues, and (**B**) two glucocorticoid responsive genes in DS. Training and testing times are indicated on each graph (see Fig. 4). Tissues were obtained one hour after testing. [N = 5/group; ^*a*^*p* < 0.05, ^*b*^*p* < 0.05]. Error bars = SEM.

Because the behavioral paradigm used in these experiments puts animals in a stress inducing situation (learning to avoid a foot shock), we questioned whether the clock gene responses found in DS and NAS might be part of a general stress response. We found no change in the expression of the glucocorticoid receptor gene (*Nr3c1*) in the DS, NAS or in amygdala (BLA, CeA) following conditioning. The amygdaloid complex was included due to its known role in mediating anxiety related behaviors^44–46^. There was also no evidence for altered activity in other genes that are sensitive to glucocorticoids (*Per1, SGK*) in either DS or NAS (Fig. 6). Therefore, we found no evidence for a glucocorticoid stress response to either conditioning or testing in the experimental or control mice. In addition, we found no evidence that cFos gene activity was altered following the stimulation that would accompany the exposure to the conditioning chamber during the testing phase in any tissue (Supplementary Fig. S8). Daily and circadian rhythms of cFos expression are found in various brain tissues and are commonly used to demonstrate the circadian responsiveness of tissue to external stimuli^47^.

### Clock resetting in a corroborative response model

We compared clock gene expression on the day following conditioning, looking specifically at gene expression at the conditioning (matching) time and expression at a non-matching time (8 hours different), when mice are not expected to exhibit the conditioned avoidance behavior. The model (described in Fig. 1) is based on the notion that a shift in an underlying molecular clockwork produces predictable changes in expression of a single gene (state variable) throughout its cycle. Furthermore, alterations of one state variable (gene transcript) are corroborated by predictable phase shifts and expression changes in other state variables. Theoretically, the phase of a system can be determined with measurements at as few as two points (Fig. 1); however, accuracy increases with additional time points and/or the assessment of more variables. Phase shifts of an oscillation can be determined by fitting its known or estimated characteristics to the new measured values. In the experiments described here, we used two test points and two genes as corroborating measurements.

**Figure 7:**
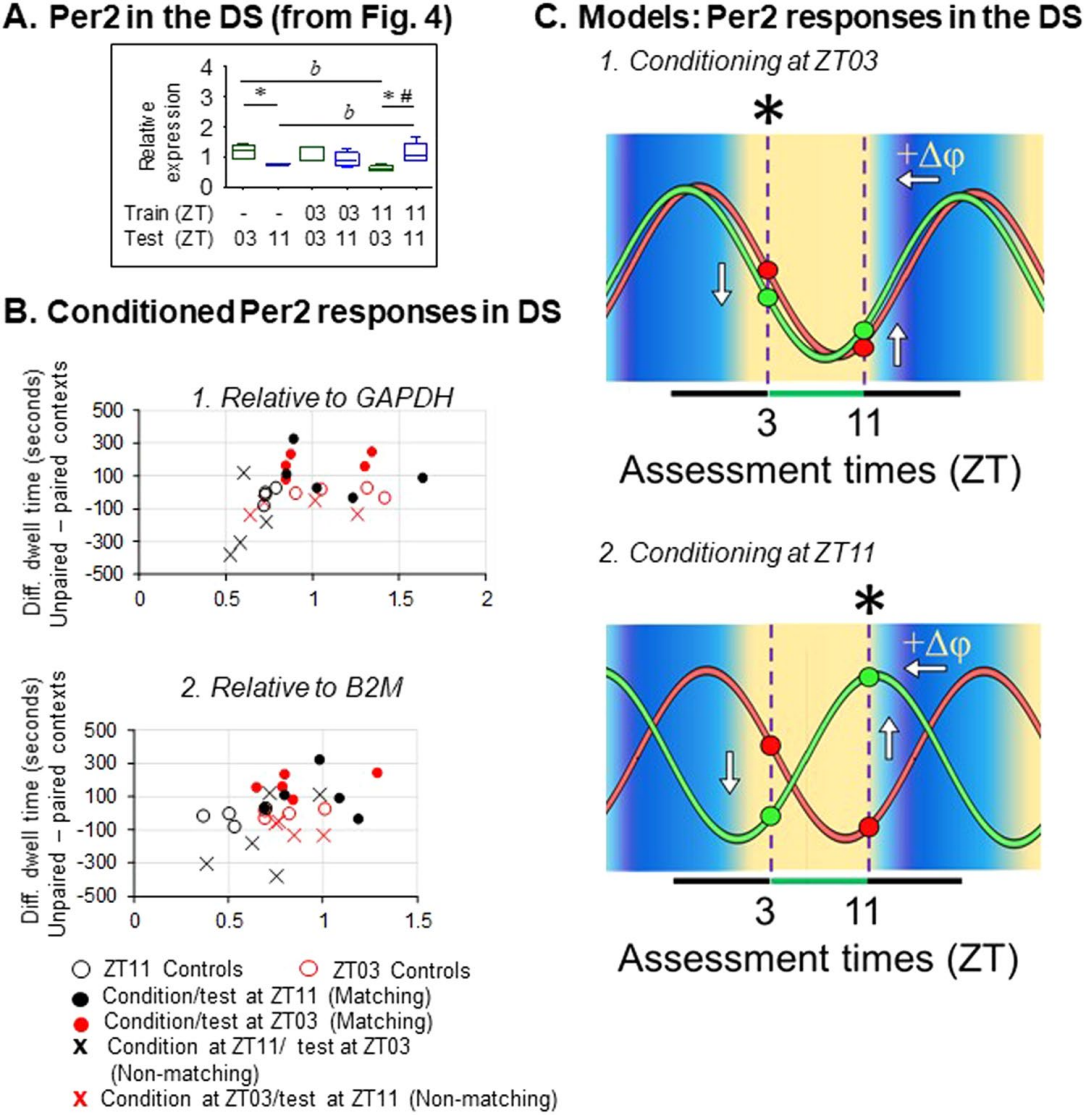
Time memory and mPer2 rhythms in the DS. (**A**) Response of *Per2* mRNA in the DS (cf. Fig. 4B, DS, Per2) [^*b*^*p* < 0.05 significant change following conditioning at ZT11; ^*^p < 0.05 phase dependent difference between ZT03 and ZT11 ^#^*p* < 0.001 significant change in phase dependent gene expression following conditioning at ZT11]. (**B**) Scatter plots of samples from individual mice. X- axis: Per2 abundance (OD) relative to (1) GADPH or (2) B2M. Y-axis: Context preference was calculated as total dwell time in unpaired (safe) minus paired (foot shock) contexts. Data shown in B1 are the same as in Panel A. Marker color indicates test time: Red = ZT03, Black = ZT11. Positive values indicate comparative preference for the safe chamber. Group statistical comparison are shown in Panel A. (**C**) Suggested *Per2* phase shifts based on the model presented in Fig. 1. ∆φ = +1.0 hr for conditioning at ZT03, and +9 to +10 hours for conditioning at ZT11. Both are estimated phase shifts required to account for differences in gene expression following conditioning. Eight-hour difference in estimated phases following the shift account for the temporal difference in conditioning times.

We examined the *Per2* results from the DS as the most likely site to verify circadian resetting accompanying time memory (Fig. 7A). As shown in Table 1, conditioning at ZT11 produced highly significant changes in the phase dependence of *Per2* expression and N/P. However, at ZT03 conditioning significantly altered N/P phase dependence, but did not alter *Per2* alone. This is represented in Fig. 7B, which shows preference score as a function of *Per2* expression for all experimental groups. Overall, higher difference scores are correlated with higher *Per2* expression using either *GAPDH* or *B2M* as reference genes; however the correlation is not significant using *GAPDH* as the reference (r = 0.368; p = 0.0643), but is significant using *B2M* (r = 0.397; *p* = 0.0446) (Fig. 7B). Therefore, CPA at matching times are linked significantly with changes in *Per2* expression in the DS and changes in N/P. *Per2* values for unconditioned controls fall into a narrow band near zero avoidance, and segregate with the ranges for conditioned animals assessed at non-matching times. A hypothetical *Per2* oscillation, based on published data from dopamine target regions^31–37,48^ and our own data (Fig. 2A) was fitted by eye to the measured control values at ZT03 and ZT11. The identical oscillation was then fitted to the *Per2* data following both conditioning events (Fig. 7C). The phase relationship between the two shifted oscillations can account for the predicted eight hour difference in time of day learned by the two conditioned groups.

## Discussion

The behavioral results from this study are consistent with previous reports that memory of an experience includes memory for the time of day (time memory), and that animals anticipate future recurrence of the conditioned context near the time of the original encounter^5–7^. As expected, conditioned animals exhibited avoidance responses to a context paired with a foot shock only at the time of day that prior conditioning had taken place. Despite eight days of place avoidance conditioning, the DS and NAS were the only brain areas where there was a response that could be interpreted as the conditioned resetting of a circadian clock gene. Clock genes in other dopamine target areas and the SCN exhibited a few isolated differences in expression levels compared with non-conditioned controls, but no evidence for clock resetting was present according to our criteria. In the aggregate the results reported here are consistent with various studies indicating that a circadian molecular clock is not required in many experimental situations that require animals to remember the time of day that important conditions were experienced. This includes implicit and explicit time memory^14–17,49^ as well as scheduled food anticipation^50–54^. The conclusion appears to be contrary to results of previous fear conditioning^33,34^ and food entrainment^35–37^ studies where the conditioning time is associated with tissue wide resetting of clock genes in regions that are included in the current study. A possible explanation for the difference may lie in the enormity of the conditioning experience. A strong stimulus, which might represent threats to survival, such as food shortage, predation threat, or injury, might result in resetting of clocks in all cells involved in processing memory of the event. Whereas, for less threatening conditions as might be encountered in natural settings several times daily, it seems likely that the discriminative power necessary for an animal to remember multiple time-place associations would be compromised if each memory involved tissue wide clock resetting. In addition, a system that is rapidly reset by sensory input (e.g. in one trial learning^10–14,16^), and is also sensitive to entraining signals from the SCN^7^, is not likely to be solely responsible for long term retention of a time memory. Therefore, tissue wide resetting may be reserved for short term anticipation of conditions involving life threatening danger or need.

On the other hand, in the DS and NAS the responses of *Per2* following conditioning fit the expected characteristics of a clock gene oscillation that had been phase shifted. In these tissues, conditioning at ZT11 was followed by altered *Per2* expression at ZT11, with altered expression appearing also at the ZT03 test time (Fig. 4). This produced a highly significant change in the relative expression of *Per2* between ZT03 and ZT11 (Table 1). These effects were not reflected in an accompanying main effect on *Nr1d1*. However, conditioning at ZT03 produced a large change in the relative expression of *Nr1d1* between the two test times (Table 1). Although the effect on *Nr1d1* was not statistically significant by itself, the N/P ratio was significantly altered by changes in *Nr1d1* following conditioning at ZT03, and by changes in *Per2* following conditioning at ZT11. Unfortunately the low amplitude of expression rhythms may have obscured a corroborating main effect on another clock gene, *Bmal1*. Nonetheless, alterations in the comparative B/P ratios at ZT03 and ZT11 following conditioning support the notion that altered *Bmal1* expression contributes to the change in B/P phase dependence.

Although the *Nr1d1* data provide some support for conditioning induced phase resetting in the striatum, the strongest evidence comes from the responses of *Per2* in the DS where conditioning was linked to changes in phase dependent expression. Because conditioning at ZT03 produced different results from conditioning at ZT11, we applied the *Per2* data to the model described in Fig. 1. *Per2* expression in DS at ZT11 was higher than controls following conditioning at ZT11, but was similar following conditioning at ZT03. Importantly, expression following conditioning at ZT11 was lower than controls at the non-matching time, and again similar following conditioning at ZT03 (Fig. 7B). Using the model, an idealized reference profile for *Per2* based on published data was fitted to the control data and then shifted to fit the measured post-conditioning values from the ZT03 and ZT11 experiments. By applying our experimental results to the model, we found that the post-conditioning data predict relative phase resetting of *Per2* rhythms that are expected from the two conditioning times. We emphasize here that these results describe relative phase, not absolute phase. Interpretation of phase shifts depends on confidence in the waveform and timing of the unperturbed control oscillation relative to ZT. The shifted curves occupy positions eight hours apart because they are fitted based on our conditioning times. However, the model also gives an indication of how the oscillation may shift when stimulated at different times. Similar concepts for detecting phase have been presented recently by Laing *et al*.^55^ using a partial least square regression method and by Anafi *et al*.^56^, using an algorithm (cyclic ordering by periodic structure; CYCLOPS), to show that circadian phase can be interpreted in a single sample from a collection of cycling transcripts. It may prove difficult to measure phase shifts using some of these methods because as our data show, not all clock gene transcripts react in the same way (or at all) to stimulation. However, the inferred circadian phase and phase shifts from expected alterations in rhythmic cell components may provide some insight into mechanisms underlying the retention and expression of time memory.

It is unlikely that the absence of detectable phase shifts in a single clock gene is due to an insufficient quantitative difference between gene expressions at the two *Zeitgeber* or circadian times. The ISH data shown in Fig. 2 indicate that differences in expression levels at test times that are eight hours apart are sufficient to detect any differences in resetting to the two conditioning times. Nevertheless, we considered the possibility that even an eight hour difference between two time points on these curves may not guarantee large changes in clock gene expression. In our experiments, we measured the expression of three genes at two testing times separated by eight hours, matching the behavioral test times. In control animals, baseline mRNA levels for *Nr1d1*, *Per2, and Bmal1* were significantly different between ZT03 and ZT11. This was reproduced multiple times using ISH and qRT-PCR methods (Fig. 2). Furthermore, expression ratios were similar for naïve and procedural (non-conditioned) control groups at each testing time. This includes the expected inverse phase relationships of mRNA oscillations in the SCN compared with all other brain regions. Therefore, the methods are sufficiently discriminative to detect phase dependent expression differences, and should reveal the shift of the clock either by alterations in single gene expression or changes in expression ratio (Fig. 4).

Our findings point to a unique role of the DS in time memory. The DS plays dual roles in the processing of goal-directed behavior governed by neural representations of relationships between responses and outcomes (R-O associations) and in the formation of habitual behaviors reflecting relationships between stimulus and response (S-R associations). The two roles generally are attributed respectively, to the dorsomedial striatum (DMS) and dorsolateral striatum (DLS)^57–60^. Together with the ventral striatum, hippocampus and cortical structures, the DS is an integral part of reward-guided decision-making and control of action. Several groups have reported associations between DS dopamine activity and circadian regulation of locomotor behavior^48,61–65^. It seems reasonable that a circadian oscillator in the DS could provide a conditioned temporal gate that is set to the time of an important event, or could amplify rhythmic responses that are driven by an oscillator located elsewhere. The DS could modulate the likelihood that a conditioned response will occur based on the phase relationship of the DS oscillator with other tissues involved in sensory-motor integration. In either arrangement, the DS may differentially favor conditioned responses at the appropriate time of day or cycle.

Within the DS, the specific response of *Per2* suggests a potential for independent and tissue specific functions of clock genes. Clock genes interact to produce circadian molecular oscillations; however, to function as a clock, the organization of clock genes must be responsive to *Zeitgeber* input, and some of the products of clock genes (the “hands” of the clock) must interact with other cell components. This leaves open the possibility that some clock genes might be influenced by both circadian regulation as well as external regulation via cellular responses. Several non-circadian roles for clock genes have been reported in mammals and Drosophila^65–71^, and dual regulation of *Per* genes has been reported in peripheral tissues in the mouse^72,73^. Therefore, temporal conditioning could affect the rhythmic expression of one (or more) clock genes without resetting the entire core clock mechanism. Already the DS has been implicated in food anticipation, which under natural circumstances requires the processing of both R-O and S-R associations. Therefore, the DS may act as a gateway for the time dependent regulation of goal directed behavior. The clock gene *Per2* in particular may participate in the conditioned regulation of behavioral responses functioning as an immediate-early response gene. Importantly, this does not preclude a more general function of the circadian molecular clock either in responding to threatening situations, or in organizing general behavioral activity according to the time of day. The results are consistent with previous reports that individual clock genes may respond independently and uniquely, suggesting historic functions for specific clock genes that are independent of their roles in circadian regulation^74–76^.

## Methods

### Animals

Both wild type C57Bl6 and mPer2^Luc^ reporter mice on a C57Bl6 background were used in these experiments. Reporter mice were included for *ex vivo* bioluminescence assessments of circadian phase in a related study not reported here. mPer2^Luc^ and C57Bl6 mice exhibited no differences in responses to conditioning in preliminary studies (see Results).

For conditioning experiments, 40 adult male mPer2^*Luc*^ mice were obtained from an inbred breeding colony at the Biological Sciences Facility (BSF) at the University of Toronto. The original breeding stock was obtained from Jackson Laboratories. For activity recording, animals were singly housed in polypropylene cages with free access to a running wheel. Food and water were available ad libitum. In all experiments, animals were entrained to a 24-h light-dark cycle maintained at 12 h light and 12 h dark (LD12:12). The time of the lights on was designed as ZT0. Cage changes were made every 10–14 days to minimize disturbance of the behavioral recordings. An independent cohort of animals was maintained at the Institute of Physiology of the Czech Academy of Sciences, Prague, CZ, that were used to provide a baseline of clock gene expression, for comparison with the conditioning experiments. Adult male C57Bl6 mice (n = 35) were maintained under LD12:12 in a temperature-controlled facility at 23 ± 2 °C with free access to food and water.

### Experimental groups

The experimental cohort was partitioned into three groups – two control groups and one conditioned. The two control groups were (a) “naïve animals” which were taken directly from the breeding colony without exposure to the conditioning apparatus; and (b) “procedural controls” that were exposed to the CPA apparatus, then were tested after 8 days (at either ZT03 or ZT11) (n = 4 at each time point). The “conditioned animals” were trained on the CPA paradigm (see below) at one of the two times of the day, ZT03 or ZT11, for 8 days and then tested for preference at one of the two times (n = 5 at each time point). All animals were anesthetized one hour after testing (ZT04 or ZT12), and sacrificed via decapitation under isoflurane anesthesia. Additionally, control C57Bl6 mice (naïve with no exposure) were sacrificed every 4 h over a 24 h period (5 mice at each time point) by decapitation in anesthesia (i.p. injections of thiopental, 50 ml per kg), and brain tissue was immediately frozen in dry ice and stored until sectioning.

### Conditioned Place Avoidance (CPA) paradigm

Conditioning was conducted in three steps: Step 1- *Pre-exposure*: Animals were pre-exposed to identical Plexiglas chambers, which only differed by the pattern on their walls. They were allowed to access both chambers through a connecting alley in order to ensure prior neutrality of the contexts for a total of 10 minutes. Step 2 - *Conditioning*: Conditioning was conducted at two times of day, ZT03 and ZT11, so that all procedures were conducted in the light. On Day 1, each animal was confined to one of the chambers for 10 minutes at a pre-determined time (either ZT03 or ZT11). One of the chambers was assigned as the shock-paired chamber for each animal; subjects received three 0.3 mA foot shocks during each 10 minute trial. The next day, at the same time, the animal was confined to the other chamber. This pattern was repeated 4 times, so that animals were exposed to each chamber 4 times over eight consecutive days. Step 3- *Testing*: On the day after conditioning, animals were placed in the alley at either one of the two test times (ZT03 or ZT11), and given access to both chambers. Dwell time in each chamber was recorded to assess presence of avoidance behavior.

### Laser capture microdissection

Coronal sections of frozen brain tissue were made at thicknesses of 20 or 30 μm. Thicker sections were sampled from larger brain nuclei. Sections containing the following regions of interest were selected: SCN, Ci, DS, NAS, NAC, sub-regions of the dorsal hippocampus (DG, CA3, CA1), amygdala (CeA, BLA). Sections were stained with cresyl violet (Sigma Aldrich, St. Louis, USA) and dissected using a laser microdissector (LMD6000, Leica), as described previously^77^. Samples from each brain area were collected individually in microfuge tubes containing RLT buffer from the RNeasy Micro kit (Qiagen, Valencia, USA) and stored until RNA isolation.

### RNA isolation and real-time PCR (qRT-PCR)

Total RNA was isolated using the RNeasy Micro kit (Qiagen, Valencia, USA) according to the manufacturer’s instructions. Isolated RNA samples were reverse-transcribed into cDNA using the SuperScript VILO cDNA Synthesis Kit (Invitrogen, Carlsbad, USA). The cDNA samples were analyzed by real-time PCR on a ViiA7 Real-Time PCR System (Life Technologies, Carlsbad, USA) using 5x HOT FIREPol Probe qPCR Mix Plus (Solis Biodyne, Tartu, Estonia) and TaqMan Gene Expression Assays (Life Technologies) specific for mouse gene *Period2* (Per2) (assay ID Mm00478113_m1), *Nr1d1* (cat. no. Mm00520708_m1), and *c-fos* (assay ID Mm00487425_m1). *Bmal1* (assay ID Mm00500226_m1) was measured only in the SCN, DS and CA1. The assay probes span exon junctions. RNA quantities were normalized using two reference genes, glyceraldehyde-3-phosphate dehydrogenase (GAPDH; assay ID Mm99999915_q1) and beta-2-microglobulin (B2M; assay ID Mm00437762_m1) (Applied Biosystems, Foster City, USA). A single PCR reaction was performed in a final volume of 14 μl. Target gene probes were dye-labeled with FAM (6-carboxyfluorescein). The GAPDH and B2M probes were labeled with VIC (4,7, 29-trichloro-79- phenyl-6-carboxyfluorescein) fluorescent dyes. The ΔΔCt method was used for the quantification of relative cDNA concentration.

### *In situ* Hybridization (ISH)

Alternate brain sections from naïve controls were processed for ISH. The cDNA fragments of mouse *Per2* (1512 bp) and *Nr1d1* (1109 bp) were used as templates for *in vitro* transcription of complementary RNA probes. Probes were labeled using ^35^S-UTP, and the ISH was performed as previously described^78^. After the hybridization (*Nr1d1* (20 h in 60 °C) and *Per2* (20 h in 61 °C) and post hybridization wash, slides were exposed to BIOMAX MR film (Kodak) for 10 days and developed using the ADEFO-MIX-S developer and ADEFOFIX fixer (ADEFO-CHEMIE Gmbh, Germany). Autoradiographs of sections were analyzed using the image analysis system (ImageJ) to detect the relative optical density (OD) of the specific hybridization signal. Each measurement was corrected for nonspecific background by subtracting OD values from the same adjacent area for each structure. The background signal of that area served as an internal standard. Finally, slides were counterstained with cresyl violet (Sigma Aldrich, St. Louis, USA) to aid with the OD analysis. The relative ODs of each animal were calculated as a mean of relative ODs of the left and right sides of each structure. Copies of images are available from the authors on request.

### Statistics and data analysis

Place preference or avoidance is based on the differential dwell time in each of the two contexts that the animals were given to compare. The neutrality of two contexts (=no preference) is assumed when there is no statistical difference between dwell times in each context when animals are given a free choice to explore, with no prior experience. The post-conditioning preference is a significant difference between the relative dwell times in the two contexts, when the animals are given a second choice trial. Dwell time begins when an animal places both forepaws on the context chamber floor, and ends when both forepaws are placed back into the connecting alley. Multiple entries and exits are summated for each of the contexts. Dwell time spent in shock vs. safe chambers was used to analyze correlations between the behavioral data and relative clock gene expression and expression ratios. Descriptive statistical analysis and quantitative analyses were performed with GraphPad Prism. Both behavioral and molecular data were analyzed using planned comparisons and unpaired t tests with Welch’s corrections, and verified with ANOVA to compare effects of conditioning on expression at ZT03 and ZT11. To quantify possible phase shifts in a single gene, difference scores were derived from measured values at the two test times for each condition. These were compared using t-tests and the outcomes verified using a Mann-Whitney U test on rank ordered and randomly paired data.

### Ethical approvals

All procedures were approved by either (a) the University of Toronto Animal Care Committee according to the guidelines of the Province of Ontario and the Canadian Council on Animal Care (CCAC); or (b) the Animal Care and Use Committee of the Institute of Physiology of the Czech Academy of Sciences and were in agreement with the Animal Protection Law of the Czech Republic, as well as the European Community Council directives 86/609/EEC.

## Electronic supplementary material

Supplementary material

## Data Availability

Original data files and images are available from the authors on request.

## References

[1] Pahl M, Zhu H, Pix W, Tautz J, Zhang S (2007). Circadian timed episodic-like memory - a bee knows what to do when, and also where. Journal of Experimental Biology.

[2] Moore D, Doherty P (2009). Acquisition of a time-memory in forager honey bees. Journal of Comparative Physiology A. Neuroethol Sens Neural Behav Physiol..

[3] Moore D, Van Nest BN, Seier E (2011). Diminishing returns: the influence of experience and environment on time-memory extinction in honey bee foragers. Journal of Comparative Physiology A. Neuroethol Sens Neural Behav Physiol..

[4] Barrett MC, Sherry DF (2012). Consolidation and reconsolidation of memory in black-capped chickadees (Poecile atricapillus). Behavioral Neuroscience.

[5] Cain SW, Chou T, Ralph MR (2004). Circadian modulation of performance on an aversion-based place learning task in hamsters. Behavioral Brain Research.

[6] Ralph MR (2002). The significance of circadian phase for performance on a reward-based learning task in hamsters. Behavioral Brain Research.

[7] Ralph MR, Sam K, Rawashdeh OA, Cain SW, Ko CH (2013). Memory for time of day (time memory) is encoded by a circadian oscillator and is distinct from other context memories. Chronobiology International.

[8] Valentinuzzi VS (2008). Memory for time of training modulates performance on a place conditioning task in marmosets. Neurobiology of Learning and Memory.

[9] Cain SW, McDonald RJ, Ralph MR (2008). Time stamp in conditioned place avoidance can be set to different circadian phases. Neurobiology of Learning and Memory.

[10] Kamin LJ (1957). The retention of an incompletely learned avoidance response. Journal of Comparative Physiology and Psychology.

[11] Holloway FA, Wansley R (1973). Multiphasic retention deficits at periodic intervals after passive-avoidance learning. Science.

[12] Holloway FA, Wansley R (1973). Multiple retention deficits at periodic intervals after active and passive avoidance learning. Behavioral Biology.

[13] Wansley R, Holloway FA (1975). “Multiple retention deficits following one-trial appetitive training. Behavioral Biology.

[14] Cain SW, Ralph MR (2009). Circadian modulation of conditioned place avoidance in hamsters does not require the suprachiasmatic nucleus. Neurobiology of Learning and Memory.

[15] Ko CH, McDonald RJ, Ralph MR (2003). The suprachiasmatic nucleus is not required for temporal gating of performance on a reward-based learning and memory task. Biological Rhythm Research..

[16] Cain SW, Chalmers JA, Ralph MR (2012). Circadian modulation of passive avoidance is not eliminated in arrhythmic hamsters with suprachiasmatic nucleus lesions. Behavioral Brain Research.

[17] Cain SW, Yoon J, Shrestha TC, Ralph MR (2014). Retention of a 24-hour time memory in Syrian hamsters carrying the 20-hour short circadian period mutation in casein kiNASe-1ε (ck1εtau/tau). Neurobiology of Learning and Memory.

[18] Lukoyanov NV, Pereira PA, Mesquita RM, Andrade JP (2002). Restricted feeding facilitates time-place learning in adult rats. Behavioral Brain Research.

[19] Wise RADA (2004). learning and motivation. Nature Reviews Neuroscience.

[20] Abercrombie ED, Keefe KA, DiFrischia DS, Zigmond MJ (1989). Differential effect of stress on *in vivo* DA release in striatum, nucleus accumbens, and medial frontal cortex. Journal of Neurochemistry.

[21] Thierry AM, Tassin JP, Blanc G, Glowinski J (1976). Selective activation of the mesocortical dopaminergic system by stress. Nature (London).

[22] Fadda F, Melis MR, Argiolas A (1978). Effect of electric foot shock on DA and 3,4-dihydroxyphenylacetic acid (DOPAC) in different brain areas of rats. Bollettino. - Societa Italiana Biologia Sperimentale..

[23] Lavielle S (1979). Blockade by benzodiazepines of the selective high increase in DA turnover induced by stress in mesocortical DAergic neurons of the rat. Brain Research.

[24] Robinson TE, Becker JB, Young EA, Akil H, Castaneda E (1987). The effects of footshock stress on regional brain DA metabolism and pituitary beta-endorphin release in rats previously sensitized to amphetamine. Neuropharmacology.

[25] Shanks N, Zalcman S, Zacharko RM, Anisman H (1991). Alterations of central norepinephrine, DA and serotonin in several strains of mice following acute stressor exposure. Pharmacology, Biochemistry and Behavior.

[26] Mistlberger RE (2011). Neurobiology of food anticipatory circadian rhythms. Physiology and Behavior.

[27] Mistlberger RE, Mumby DG (1992). The limbic system and food-anticipatory circadian rhythms in the rat: ablation and DA blocking studies. Behavioral Brain Research.

[28] Spyraki C, Fibiger HC, Phillips AG (1982). DAergic substrates of amphetamine -induced place preference conditioning. Brain Research.

[29] Carr GD, White NM (1983). Conditioned place preference from intra-accumbens but not intra-caudate amphetamine injections. Life Sciences.

[30] Cain SW, Rawashdeh OA, Siu M, Kim SC, Ralph MR (2017). Dopamine dependent setting of a circadian oscillator underlying the memory for time of day. Neurobiology of Learning and Memory.

[31] Harbour VL (2013). Comprehensive mapping of regional expression of the clock protein PERIOD2 in rat forebrain across the 24-h day. PLoS One.

[32] Chun LE, Morton S, Hinds LR, Spencer RL (2015). Variations in Phase and Amplitude of rhythmic clock gene expression across prefrontal cortex, hippocampus, amygdala, and hypothalamic paraventricular and suprachiasmatic nuclei of male and female rats. Journal of Biological Rhythms.

[33] Al-Safadi S (2014). Stress-Induced Changes in the Expression of the Clock Protein PERIOD1 in the Rat Limbic Forebrain and Hypothalamus: Role of Stress Type, Time of Day, and Predictability. PLoS One.

[34] Pantazopoulos H, Dolatshad H, Davis FC (2011). A fear-inducing odor alters PER2 and c-Fos expression in brain regions involved in fear memory. PLoS One..

[35] Wakamatsu H (2001). Restricted-feeding-induced anticipatory activity rhythm is associated with a phase-shift of the expression of mPer1 and mPer2 mRNA in the cerebral cortex and hippocampus but not in the suprachiasmatic nucleus of mice. European Journal of Neuroscience.

[36] Ángeles-Castellanos M, Mendoza J, Escobar C (2007). Restricted feeding schedules phase shift daily rhythms of c-Fos and protein Per1 immunoreactivity in corticolimbic regions in rats. Neuroscience..

[37] Verwey M, Amir S (2012). Variable restricted feeding disrupts the daily oscillations of Period2 expression in the limbic forebrain and dorsal striatum in rats. Journal of Molecular Neuroscience.

[38] Radonić A (2004). Guideline to reference gene selection for quantitative real-time PCR. Biochemical and Biophysical Research Communications.

[39] Chapman JR, Waldenström J (2015). With reference to reference Genes: A Systematic Review of Endogenous Controls in Gene Expression Studies. PLoS One.

[40] Kozera B, Rapacz M (2013). Reference genes in real-time PCR. Journal of Applied Genetics.

[41] Lee KS (2009). Validation of commonly used reference genes for sleep-related gene expression studies. BMC Molecular Biology.

[42] Cleal JK, Shepherd JN, Shearer JL, Bruce KD, Cagampang FR (2014). Sensitivity of housekeeping genes in the suprachiasmatic nucleus of the mouse brain to diet and the daily light–dark cycle. Brain Research.

[43] Jain N, Vergish S, Khurana JP (2018). Validation of house-keeping genes for normalization of gene expression data during diurnal/circadian studies in rice by qRT-PCR. Scientific Reports.

[44] Shepard JD, Barron KW, Myers DA (2000). Corticosterone delivery to the amygdala increases corticotropin-releasing factor mRNA in the central amygdaloid nucleus and anxiety-like behavior. Brain Research.

[45] Myers B, Greenwood-Van Meerveld B (2010). Elevated corticosterone in the amygdala leads to persistant increases in anxiety-like behavior and pain sensitivity. Behavioral Brain Research.

[46] Tran L, Greenwood-Van Meerveld B (2012). Altered expression of glucocorticoid receptor and corticotropin-releasing factor in the central amygdala in response to elevated corticosterone. Behavioral Brain Research.

[47] Sumová A, Trávnícková Z, Mikkelsen JD, Illnerová H (1998). Spontaneous rhythm in c-Fos immunoreactivity in the dorsomedial part of the rat suprachiasmatic nucleus. Brain Research.

[48] Hood S (2010). Endogenous dopamine regulates the rhythm of expression of the clock protein PER2 in the rat dorsal striatum via daily activation of D2 dopamine receptors. Journal of Neuroscience.

[49] Mulder C, Van Der Zee EA, Hut RA, Gerkema MP (2013). Time-place learning and memory persist in mice lacking functional Per1 and Per2 clock genes. Journal of Biological Rhythms.

[50] Pitts S, Perone E, Silver R (2003). Food-entrained circadian rhythms are sustained in arrhythmic Clk/Clk mutant mice. American Journal of Physiology, Regulatory and Integrative Comparative Physiology.

[51] Iijima M (2005). Altered food-anticipatory activity rhythm in Cryptochrome deficient mice. Neuroscience Research.

[52] Pendergast JS (2009). Robust food anticipatory activity in BMAL1-deficient mice. PLoS One.

[53] Storch KF, Weitz CJ (2009). Daily rhythms of food-anticipatory behavioral activity do not require the known circadian clock. Proceedings of the National Academy of Science, USA.

[54] Takasu NN (2012). Circadian regulation of food-anticipatory activity in molecular clock deficient mice. PLoS One.

[55] Laing EE, Möller-Levet CS, Poh N, Santhi N, Archer SN (2017). Derk-Jan Dijk. Blood transcriptome based biomarkers for human circadian phase. eLife.

[56] Anafi RC, Francey LJ, Hogenesch JB, Kim J (2017). CYCLOPS reveals human transcriptional rhythms in health and disease. Proceedings of the National Academy of Science, USA.

[57] Balleine BW, O’Doherty JP (2010). Human and rodent homologies in action control: corticostriatal determinants of goal-directed and habitual action. Neuropsychopharmacology.

[58] van der Meer MA, Johnson A, Schmitzer-Torbert NC, Redish AD (2010). Triple dissociation of information processing in dorsal striatum, ventral striatum, and hippocampus on a learned spatial decision task. Neuron.

[59] Gremel CM, Costa RM (2013). Orbitofrontal and striatal circuits dynamically encode the shift between goal-directed and habitual actions. Nature Communications.

[60] Burton AC, Nakamura K, Roesch MR (2015). From ventral-medial to dorsal-lateral striatum: Neural correlates of reward-guided decision making. Neurobiology of Learning and Memory.

[61] Iijima M, Nikaido T, Akiyama M, Moriya T, Shibata S (2002). Methamphetamine-induced, suprachiasmatic nucleus-independent circadian rhythms of activity and mPer gene expression in the striatum of the mouse. European Journal of Neuroscience.

[62] Gallardo CM (2014). Dopamine receptor 1 neurons in the dorsal striatum regulate food anticipatory circadian activity rhythms in mice. eLife..

[63] Blum ID (2014). A highly tunable dopaminergic oscillator generates ultradian rhythms of behavioral arousal. eLife.

[64] Wang Y, Zhou FM (2017). Striatal But Not Extrastriatal Dopamine Receptors Are Critical to Dopaminergic Motor Stimulation. Frontiers in Pharmacology.

[65] de Lartigue G. & McDougle M. Dorsal striatum dopamine oscillations: setting the pace of food anticipatory activity. Acta Physiol (Oxf). Jun 19:e13152, 10.1111/apha.13152. [Epub ahead of print] (2018). 10.1111/apha.13152 PMC6301124 29920950

[66] O’Brien Kylie B., Sharrief Anjail Z., Nordstrom Eric J., Travanty Anthony J., Huynh Mailee, Romero Megan P., Bittner Katie C., Bowser Michael T., Burton Frank H. (2018). Biochemical markers of striatal desensitization in cortical-limbic hyperglutamatergic TS- & OCD-like transgenic mice. Journal of Chemical Neuroanatomy.

[67] Hendricks JC (2001). A non-circadian role for cAMP signaling and CREB activity in Drosophila rest homeostasis. Nature Neuroscience.

[68] Franken P, Thomason R, Heller HC, O’Hara BF (2007). A non-circadian role for clock-genes in sleep homeostasis:a strain comparison. BMC Neuroscience.

[69] Albrecht U, Bordon A, Schmutz I, Ripperger J (2007). The multiple facets of Per2. Cold Spring Harbor Symposium on Quantitative Biology.

[70] Yelamanchili SV (2006). Differential sorting of the vesicular glutamate transporter 1 into a defined vesicular pool is regulated by light signaling involving the clock gene Period2. Journal of Biological Chemistry.

[71] Sakai T, Tamura. T, Kitamoto T, Kidokoro Y (2004). A clock gene, period, plays a key role in long-term memory formation in Drosophila. Proceedings of the National Academy of Science USA.

[72] Kornmann B, Schaad O, Bujard H, Takahashi JS, Schibler U (2007). System-driven and oscillator-dependent circadian transcription in mice with a conditionally active liver clock. PLoS Biology.

[73] Ramanathan C (2014). Cell Type-Specific Functions of Period Genes Revealed by Novel Adipocyte and Hepatocyte Circadian Clock Models. PLoS Genetics.

[74] Ogawa Y (2011). Positive autoregulation delays the expression phase of mammalian clock gene Per2. PLo.S One.

[75] Albrecht Urs, Sun Zhong Sheng, Eichele Gregor, Lee Cheng Chi (1997). A Differential Response of Two Putative Mammalian Circadian Regulators, mper1and mper2, to Light. Cell.

[76] Wijnen H, Young MW (2006). Interplay of circadian clocks and metabolic rhythms. Annual Reviews Genetics.

[77] Houdek P, Sumova A (2014). *In vivo* initiation of Clock gene expression rhythmicity in fetal rat suprachiasmatic nuclei. PLoS One.

[78] Sumova A, Jac M, Sladek M, Sauman I, Ilnerova H (2003). Clock gene daily profiles and their phase relationship in the rat suprachiasmatic nucleus are affected by photoperiod. Journal of Biological Rhythms.

