# Supplementary material for: Implicit time-place conditioning alters Per2 mRNA expression selectively in striatum without shifting its circadian clocks
Source: Sci Rep. 2018 Oct 19;8:15547. doi: 10.1038/s41598-018-33637-y (PMC6195625; doi:10.1038/s41598-018-33637-y)
Supplement: Supplementary file 1 — Supplementary material [file 41598_2018_33637_MOESM1_ESM.pdf]

Supplementary material

Implicit time-place conditioning alters *Per2* mRNA expression selectively in striatum without shifting its circadian clocks

Tenjin C. Shrestha[1], Karolína Šuchmanová[2], Pavel Houdek[2], Alena Sumová[2], &

Martin R. Ralph[3*]

[1]Department of Cell and Systems Biology, University of Toronto, Toronto, Ontario, Canada.

[2]Department of Neurohumoral Regulations, Institute of Physiology of the Czech Academy of Sciences, Prague, Czech Republic.

[3] Department of Psychology, University of Toronto, Toronto, Ontario, Canada.

*Correspondence: Martin R. Ralph
Phone: (416) 978-7621
email: ralph@psych.utoronto.ca
Fax: (416) 978-4811

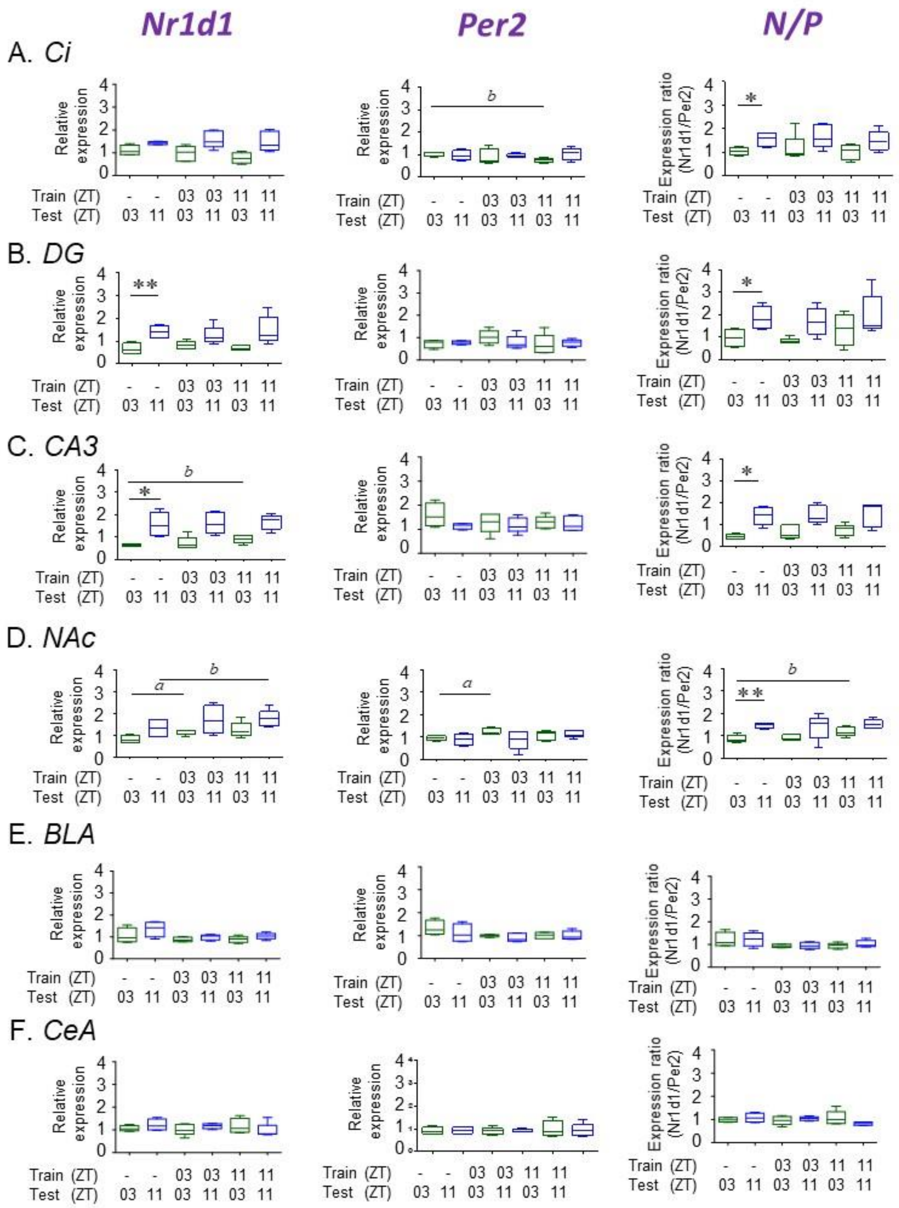

Supplementary Figure S1. Effects of behavioral conditioning on temporal gene expression. *Nr1d1*, *Per2* and N/P ratio is shown in four selected tissues. Each graph represents two conditioning experiments comparing gene expression following place conditioning at either ZT03 or ZT11 with procedural controls. Animals were tested for place preference at either ZT03 or ZT11. Tissues were obtained one hour after testing. Phase dependent differences within each experimental condition (control, ZT03 conditioned, ZT11 conditioned) were evaluated using planned comparisons and t-tests [* = $p<0.05$; ** = $p<0.01$]. Effects of conditioning were evaluated using one-way ANOVAs for control vs. ZT03 conditioning [a = $p<0.05$] and vs. ZT11 conditioning [b = $p<0.05$]). Alteration in the relative expression at ZT03 vs. ZT11 determined from difference scores between the two times were not significant. *P* values for comparisons of all difference scores are shown in Table 1. Error bars = SEM.

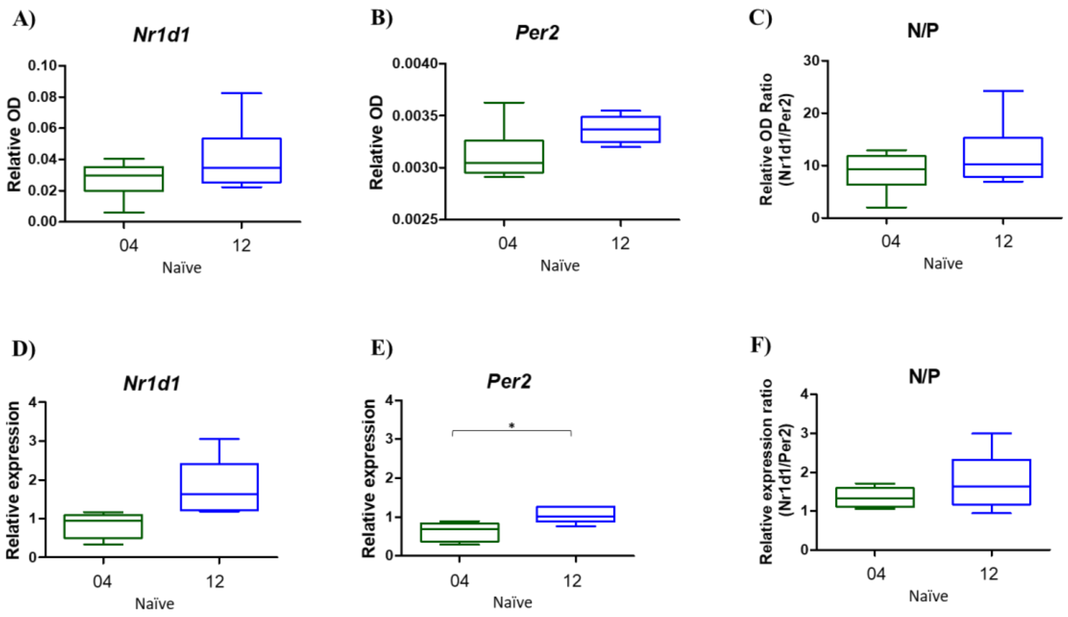

Supplementary Figure S2. Confirmation of DS data with two methods in naïve control mice. Using *in situ* (A-C) and RT-qPCR data (D-F), profiles of *Nr1d1*, *Per2* and N/P ratio in the DS were compared. N/P refers to *Nr1d1/Per2*. (* = $p<0.05$; ** = $p<0.01$). Error bars = SEM.

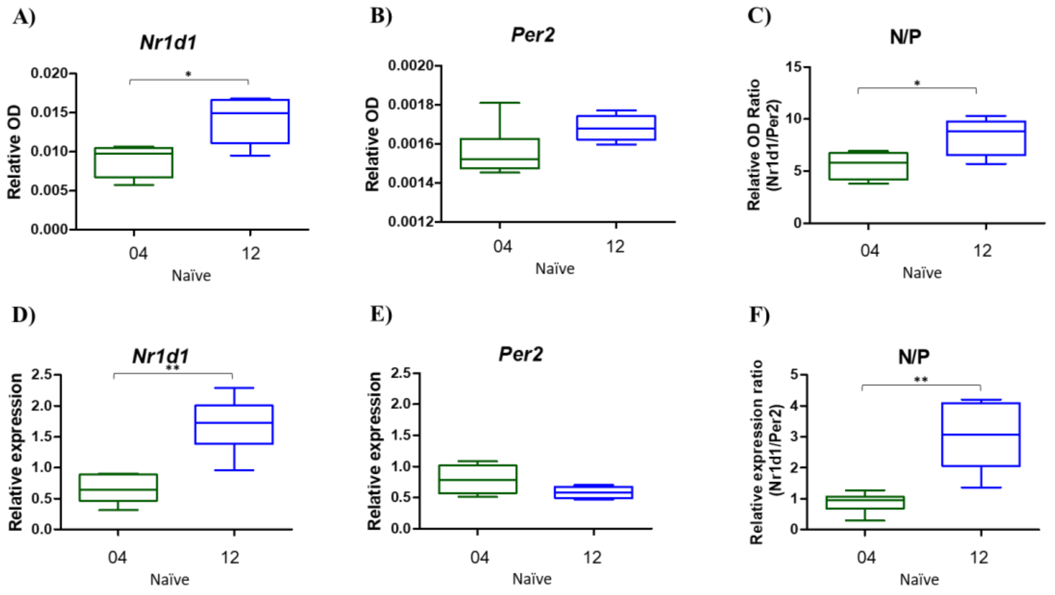

Supplementary Figure S3. Confirmation of Ci data with two methods in naïve control mice. Using *in situ* (A-C) and RT-qPCR data (D-F), profiles of *Nr1d1*, *Per2* and N/P ratio in the Ci were compared. N/P refers to *Nr1d1/Per2*. (* = $p<0.05$). Error bars = SEM.

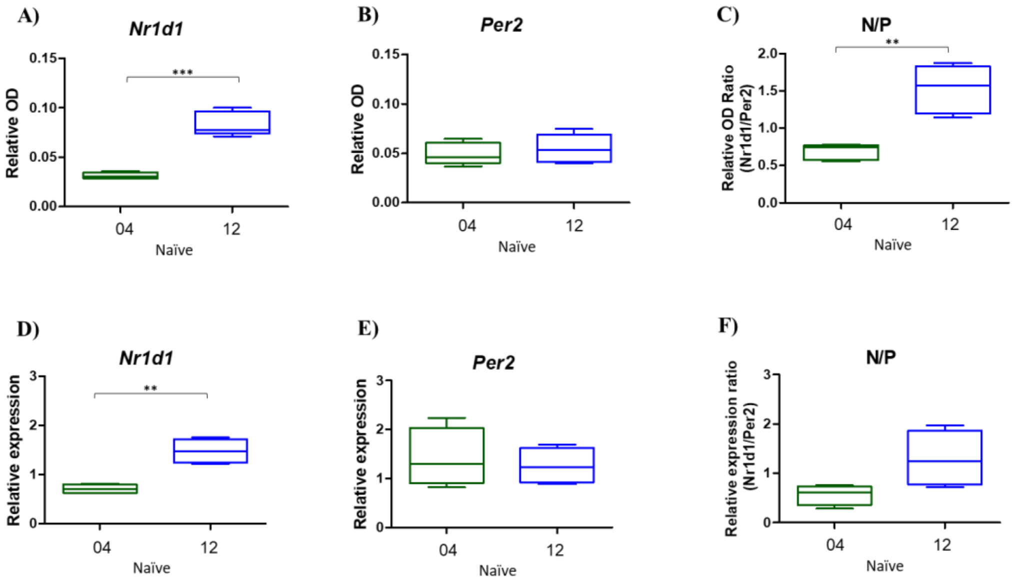

Supplementary Figure S4. Confirmation of DG data with two methods in naïve control mice. Using *in situ* (A-C) and RT-qPCR data (D-F), profiles of *Nr1d1*, *Per2* and N/P ratio in the DG were compared. N/P refers to *Nr1d1/Per2*. (* = p<0.05; ** = p<0.01). Error bars = SEM.

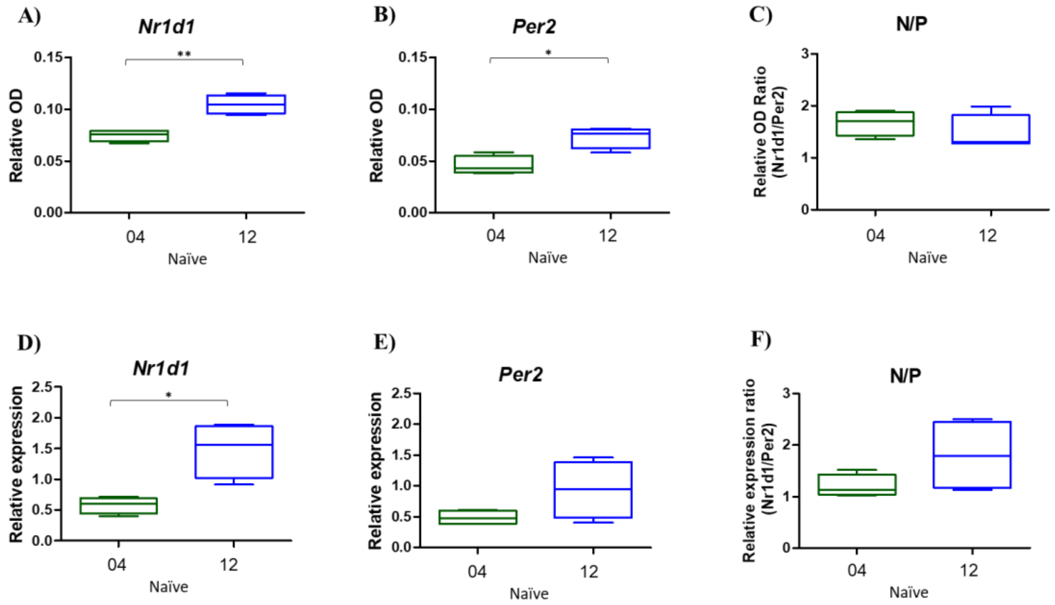

Supplementary Figure S5. Confirmation of CA3 data with two methods in naïve control mice. Using *in situ* (A-C) and RT-qPCR data (D-F), profiles of *Nr1d1*, *Per2* and N/P ratio in the CA3 were compared. N/P refers to *Nr1d1/Per2*. (** = p<0.01, ***=p<0.001). Error bars = SEM.

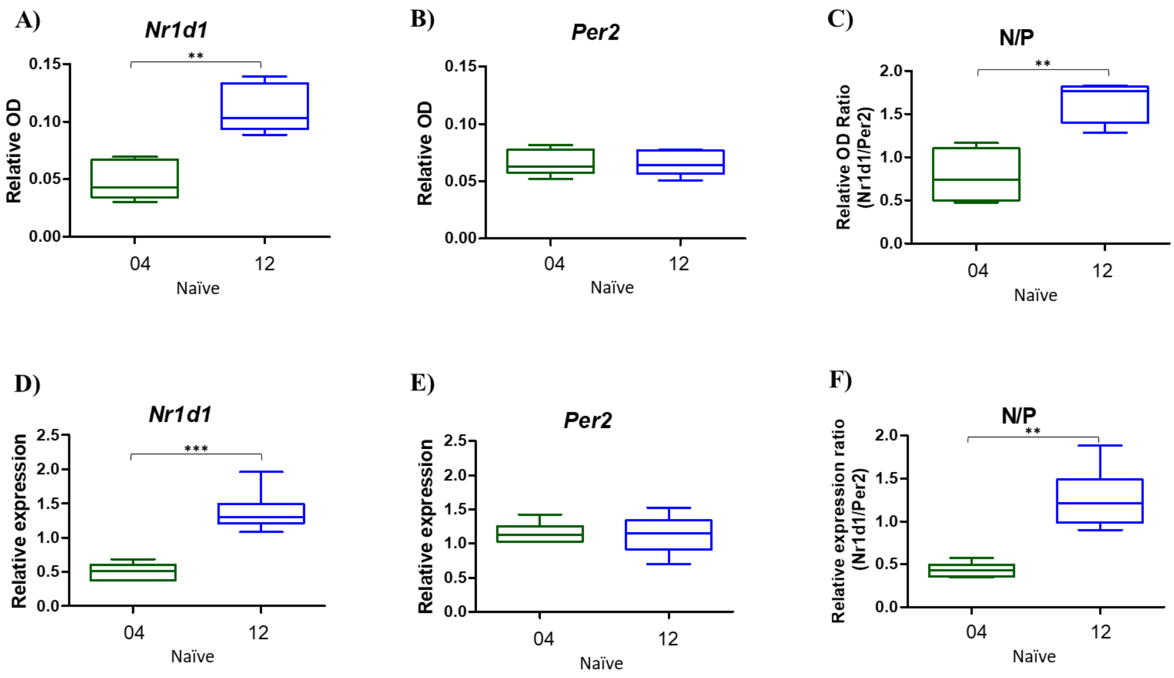

Supplementary Figure S6. Confirmation of CA1 data with two methods in naïve control mice. Using *in situ* (A-C) and RT-qPCR data (D-F), profiles of *Nr1d1*, *Per2* and N/P ratio in the CA1 were compared. N/P refers to *Nr1d1/Per2*. (** = $p<0.01$, ***=$p<0.001$). Error bars = SEM.

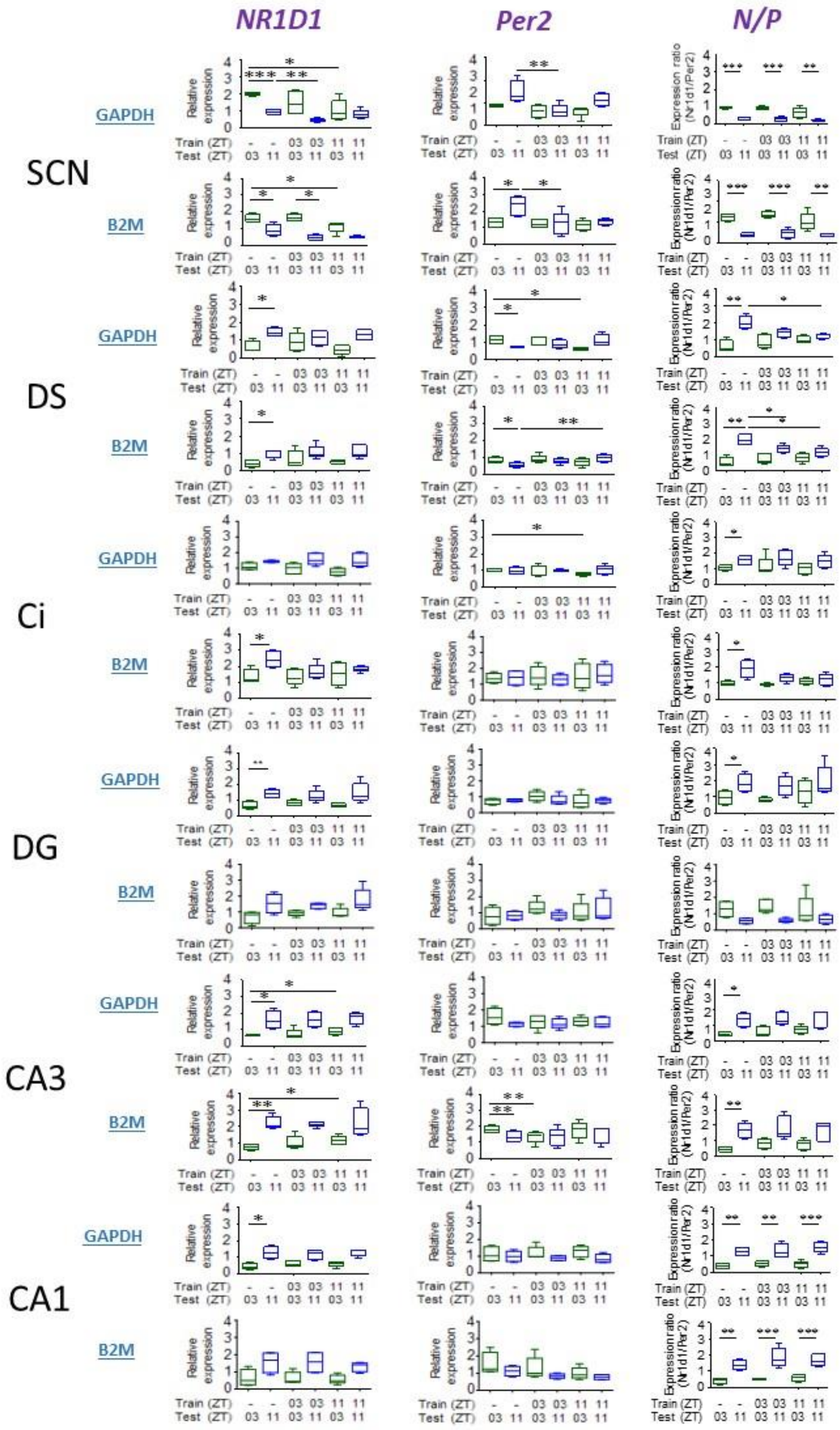

(Supplementary Figure S7)

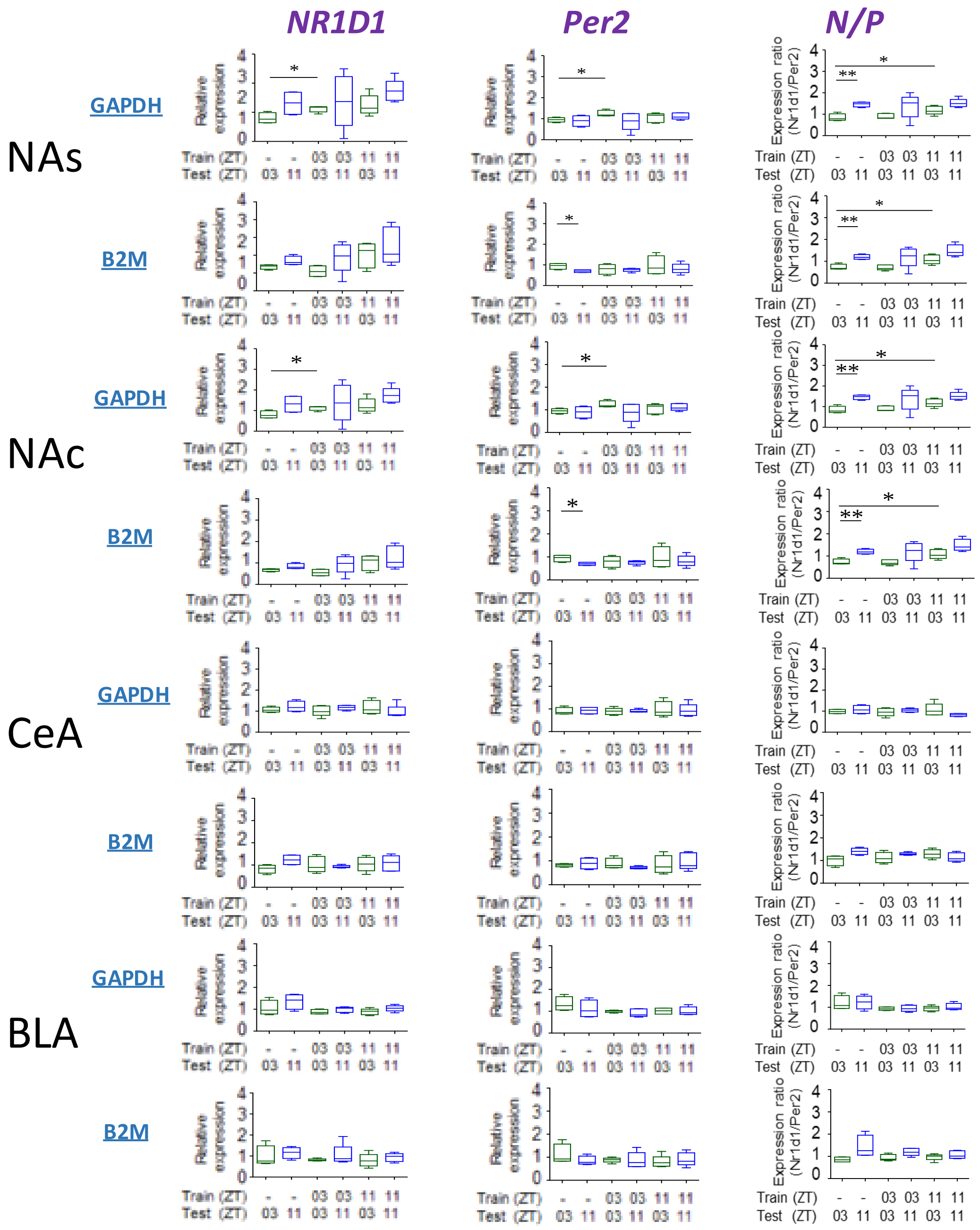

Supplementary Figure S7. Effect of behavioral conditioning on temporal expression of *Hr1d1* and *Per2*: comparison of two reference HKGs. Training and testing times are indicated on each graph (see Fig.4). [* = p<0.05; ** = p<0.01, *** = p<0.001]. Error bars = SEM.

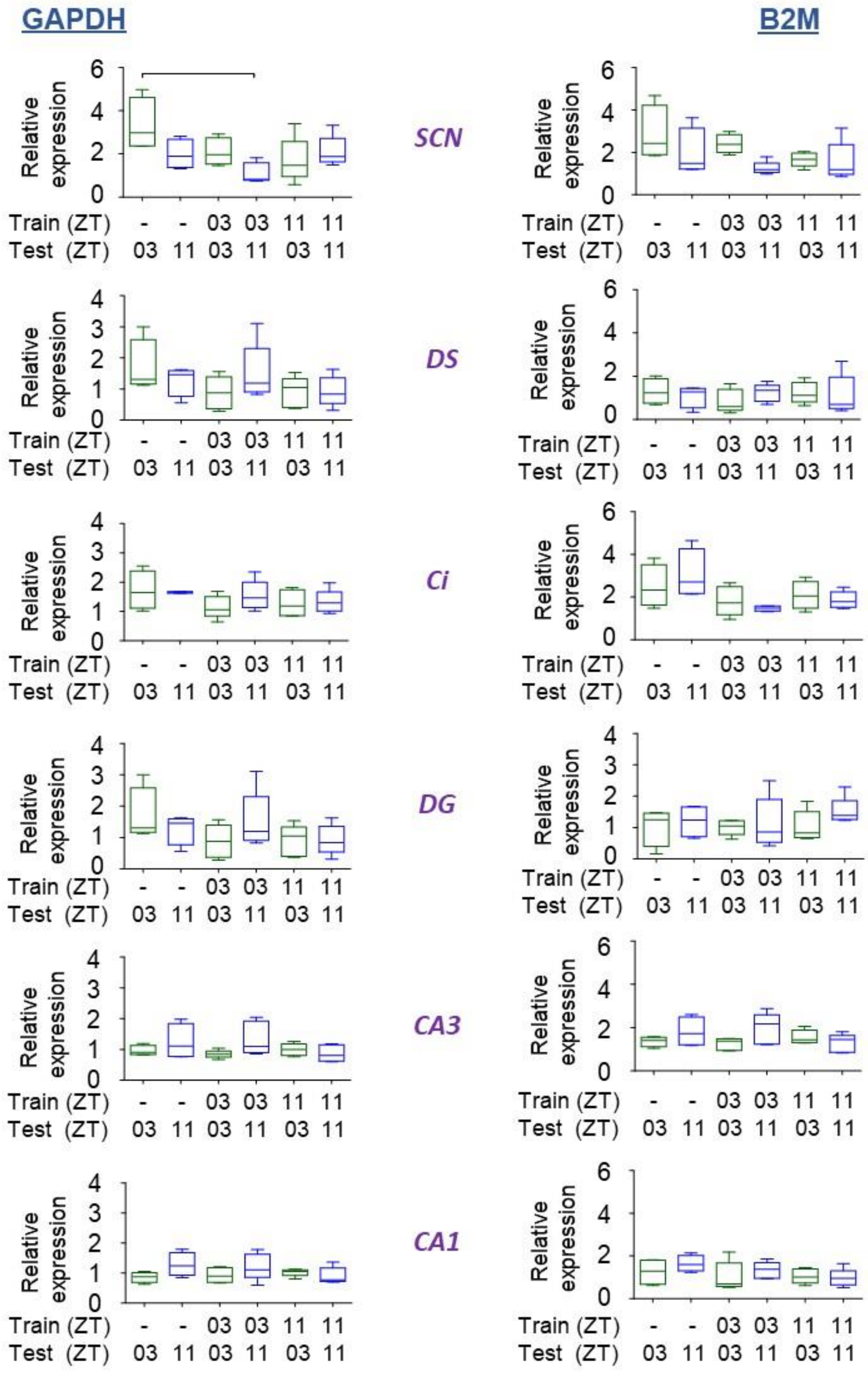

Supplementary Figure S8. Effect of behavioral conditioning on temporal expression of *cFos* relative to two HKGs. Training and testing times are indicated on each graph (see Fig.4). [* = $p<0.05$; all other comparisons *n.s.*]. Error bars = SEM.
